# Management of advanced gastric cancer: An overview of major findings from meta-analysis

**DOI:** 10.18632/oncotarget.12102

**Published:** 2016-09-17

**Authors:** Xiaolong Qi, Yanna Liu, Wei Wang, Danxian Cai, Wende Li, Jialiang Hui, Chuan Liu, Yanxia Zhao, Guoxin Li

**Affiliations:** ^1^ Department of General Surgery, Nanfang Hospital, Southern Medical University, Guangzhou, China; ^2^ Department of Gastric and Pancreatic Surgery, Sun Yat-sen University Cancer Center, Guangzhou, China; ^3^ Guangdong Laboratory Animal Monitoring Institute, Guangzhou, China; ^4^ Cancer Center, Union Hospital, Tongji Medical College, Huazhong University of Science and Technology, Wuhan, China

**Keywords:** advanced gastric cancer, management, surgery, chemotherapy, meta-analysis

## Abstract

This study aims to provide an overview of different treatment for advanced gastric cancer. In the present study, we systematically reviewed the major findings from relevant meta-analyses. A total of 54 relevant papers were searched via the PubMed, Web of Science, and Google scholar databases. They were classified according to the mainstay treatment modalities such as surgery, chemotherapy and others. Primary outcomes including overall survival, response rate, disease-free survival, recurrence-free survival, progression-free survival, time-to-progression, time-to failure, recurrence and safety were summarized. The recommendations and uncertainties regarding the treatment of advanced gastric cancer were also proposed. It was suggested that laparoscopic gastrectomy was a safe and technical alternative to open gastrectomy. Besides, neoadjuvant chemotherapy and adjuvant chemotherapy were thought to benefit the survival over surgery alone. And it was demonstrated in the study that targeted therapy like anti-angiogenic and anti-HER2 agents but anti-EGFR agent might have a significant survival benefit.

## INTRODUCTION

In global, about 952,000 cases of gastric cancer (GC) are newly diagnosed every year. As the third-leading cause of cancer related mortality, GC accounts for 841 000 deaths in 2013 globally [1, 2]. GC is early detected more commonly in Japan and South Korea, probably due to active screening programs. In China, GC is the second diagnosed cancer, incidence of which comes to 67.9% [3]. Unfortunately, more than half of radically resected GC patients relapse locally or with distant metastases, or receive the diagnosis of GC when tumor is disseminated. Thus median survival rarely exceeds 12 months, and 5-year survival is less than 10% [4]. For locally advanced gastric cancer (AGC), surgery remains the only curative therapy. The use of laparoscopic gastrectomy (LG) in AGC remains controversial because of doubts about its efficacy and safety. Perioperative and adjuvant chemotherapy (AC), as well as chemoradiation are thought to bring benefits. For unresectable AGC with overt metastatic disease, palliative chemotherapy, targeted therapy and basic supportive care (BSC) are recommended by the National Comprehensive Cancer Network (NCCN) guideline and Japanese Gastric Cancer Association (JGCA) guideline [5]. In patients with good performance status, chemotherapy is better than BSC only [4]. Though positive effect has been achieved in recent years with second- and further lines of chemotherapy, as well as using HER2-targeting drugs and ramucirumab for AGC, many phase III trials regarding the use of other targeted agents such as EGFR inhibitors or mTOR inhibitors have had negative results [6]. Thus, many questions on AGC treatment remain unresolved. In the present study, we systematically reviewed the major findings from all meta-analyses regarding the treatment of AGC and attempted to propose the evidence-based recommendations and uncertainties.

## RESULTS

**Figure 1:**
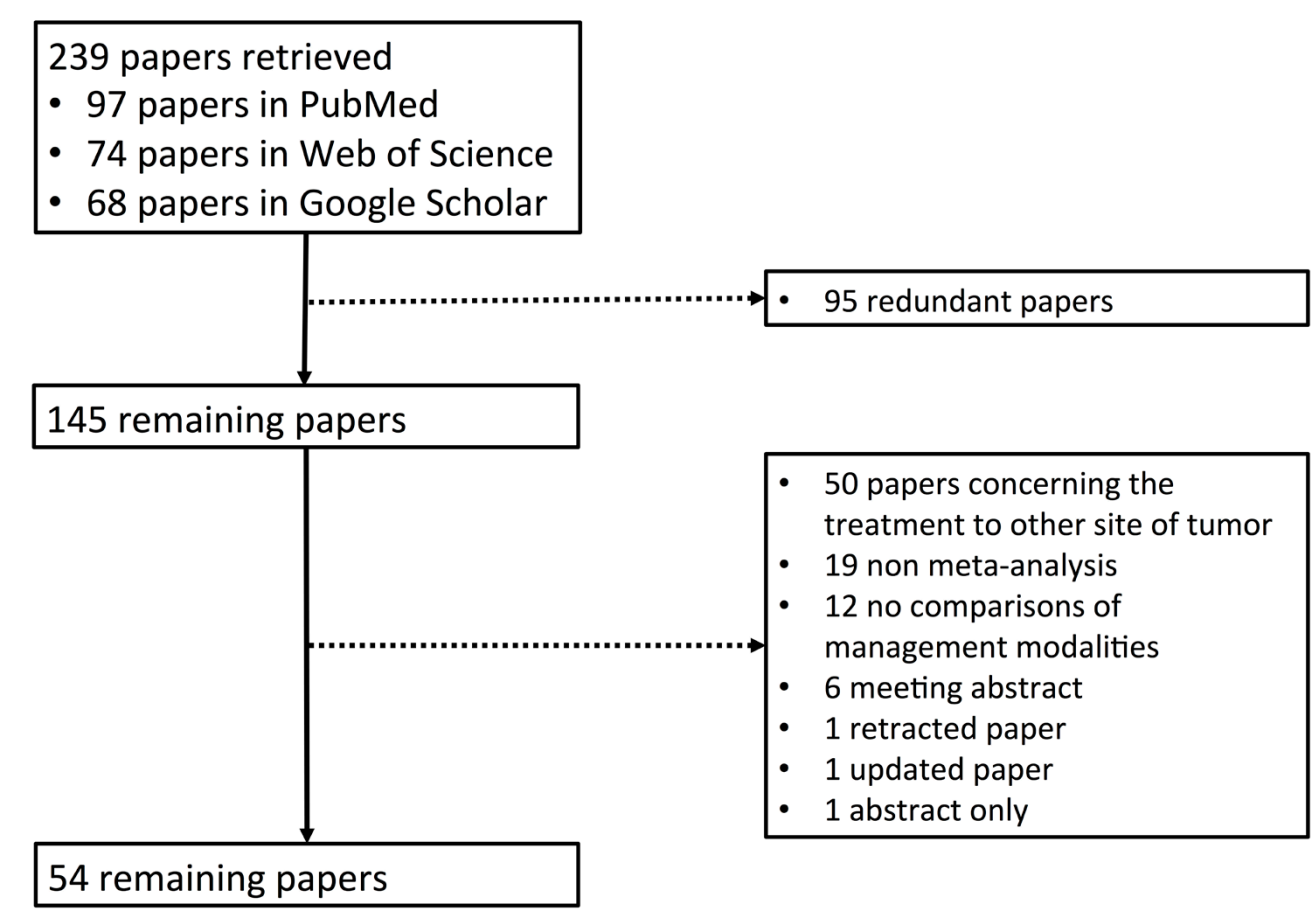
Flowchart of study inclusion

Overall, 239 papers were identified. Among them, 54 meta-analysis papers were finally included [7-60] (Figure 1). The characteristics of these included papers were shown in Supplementary Table S1. Their major findings were summarized according to the treatment modalities (Tables 1-18 and Supplementary Tables S2-S21).

**Table 1:** Findings of meta-analyses: An overview of included studies regarding LG vs. *OG*

First Author	Journal (Year)	Comparisons	OS	SR, DFS, RFS, PFS, Recurrence, TTF,TTP	Major comments
Lu C[12]	Surg Endosc(2015)	LADG vs. ODG	NA.	NA.	D2 lymphadenectomy performed laparoscopically was as effective as an open procedure in AGC.
Quan Y[13]	Gastric Cancer (2015)	LG vs. OG	OS:1-, 3-, 5-y: statistically similar.	DFS: 3-, 5-year: statistically similar. Recurrence: favor LG.	LG appeared comparable with OG in short- and long-term results.
Huang YL[17]	Int J Clin Exp Med(2014)	LAG vs. OG	OS: 3-y: statistically similar.	Recurrence: statistically similar.	LAG with D2 lymph node dissection was a feasible and safe procedure for AGC.
Zou ZH[26]	World J Gastroenterol (2014)	LGD2 vs. OGD2	OS: 3-, 5- y: statistically similar.	DFS (3-, 5- year), Recurrence/ metastasis: statistically similar.	LGD2 might be safe and effective, and offered some advantages over OGD2 for locally AGC.
Chen K[27]	World J Surg Oncol (2013)	LG vs. OG	OS (1-, 3-, 5- y), Mortality: statistically similar.	DFS (3-, 5- year), Recurrence: statistically similar.	LG was a safe technical alternative to OG with a lower complication rate and enhanced postoperative recovery.
Ye LY[38]	J Zhejiang Univ Sci B (2013)	LAG vs. OG	NA.	Recurrence: statistically similar.	LAG could be performed safely for AGC with adequate lymphadenectomy and has several short-term advantages.
Qiu J[34]	Surg Laparosc Endosc Percutan Tech (2013)	LADG vs. ODG	OS:3-y: statistically similar.	Recurrence: statistically similar.	The oncologic outcomes of LADG for AGC patients were comparable with open approach.
Choi YY[29]	J Surg Oncol (2013)	LG vs. OG	Statistically similar.	DFS: statistically similar.	There was no evidence that LG was inferior to OG.
Martinez-Ramos D[45]	Rev Esp Enferm Dig (2011)	LG vs. OG	Statistically similar.	NA.	LG was associated with a longer operative time but lower blood loss and shorter postoperative hospital stay.

**Table 2:** Findings of meta-analyses: An overview of included studies regarding regarding NAC and AC

First Author	Journal (Year)	Comparisons	OS	SR, DFS, RFS, PFS, Recurrence, TTF,TTP	Other	Major comments
**NAC vs. no chemotherapy before surgery**						
Xiao F[41]	Chongqing Medicine (2012)	Surgery with vs. without NAC	Favor NAC	NA.	Resection rate: favor NAC. Peri-operative mortality: statistically similar.	NAC could improve the tumor resection rate and the survival rate in AGC patients without increasing the operative risk.
Li W[50]	World J Gastroenterol (2010)	Surgery with vs. without NAC	Favor NAC	PFS: 3-year: favor NAC.	Tumor down-staging rate, R0 resection rate: favor NAC. Peri- operative mortality: statistically similar.	NAC could improve tumor stage and survival rate of patients with AGC with a rather good safety.
**AC vs. surgery only**						
Sun J[36]	BMC Cancer (2013)	(Include palliative gastrectomy with vs. without AC).	Favor palliative gastrectomy with AC.	NA.	NA.	Palliative gastrectomy combined with chemotherapy maight improve survival.
Sun P[57]	Br J Surg (2009)	Surgery with vs. without AC.	Favor surgery with AC.	NA.	NA.	Postoperative chemotherapy could improve OS after radical surgery for gastric cancer.

**Table 3:** Findings of meta-analyses: An overview of included studies regarding Surgery with vs. without IPC

First Author	Journal (Year)	Comparisons	OS	SR, DFS, RFS, PFS, Recurrence, TTF,TTP	Other	Major comments
Coccolini F[16]	Eur J Surg Oncol (2014)	Surgery with vs. without IPC.	OS: 1-, 2-,3-year: favor surgery+IPC; 5-year: statistically similar.	Overall recurrence, peritoneal recurrence, haematogenous metastasis: favor surgery + IPC. Lymph-nodal recurrence: statistically similar.	Mortality: 1-, 2-, 3-year: favor surgery+ IPC; 5-year: statistically similar; 2-, 3-year in patients with loco-regional nodal metastasis, 1, 2-year in patients with serosal infiltration: favor surgery+IPC. Morbidity: higher in surgery alone.	IPC had positive effect on peritoneal recurrence and distant metastasis. Morbidity rate is incremented by IPC. Loco-regional lymph-nodes invasion in patients affected by AGC was not a contraindication to IPC.
Yan TD[58]	Ann Surg Oncol (2007)	Surgery with vs. without IPC.	Favor surgery with HIIPC or with HIIPC+ EPIPC (but statistically similar between surgery with NIIPC, EPIPC or DPIPC and surgery without IPC).	Peritoneal recurrence (surgery with HIIPC or NIIPC vs. control): statistically similar.	Perioperative mortality: statistically similar. Risk of intra-abdominal abscess, neutropenia: higher in IPC+ surgery.	HIIPC with or without EPIPC after resection of AGC improved the overall survival. However, increased risk of intra-abdominal abscess and neutropenia were also demonstrated.

**Table 4:** Findings of meta-analyses: An overview of included studies regarding lymphadenectomy

First Author	Journal (Year)	Comparisons	OS	SR, DFS, RFS, PFS, Recurrence, TTF,TTP	Other	Major comments
**D2 vs. D4 lymphadenectomy**						
Wang Z[53]	World J Gastroenterol (2010)	D4 vs. D2 lymphadenectomy	OS: 5-y: statistically similar	NA.	Postoperative morbidity and mortality: statistically similar; Operation time, blood loss: favor D2 group.	D4 could be performed as safely as standard D2 dissection without increasing post-operative mortality but failed to benefit OS.
Zhang YL[54]	Chin J Gen Surg (2010)	D2 vs. D4 lymphadenectomy	NA.	SR: 5-y: Statistically similar; Recurrence: 5-y: statistically similar.	Postoperative mortality and complications: statistically similar.	The efficacy and safety of D2 and D4 lymph node dissection for AGC patients was significantly similar but have yet to be accurately analysed.
**D2 vs. D3 lymphadenectomy**						
Zhang L[48]	Chin Gen Prac (2011)	D2 vs. D3 lymphadenectomy	NA.	SR: 5-y: statistically similar	Postoperative mortality, anastomotic leakage, pneumonia: statistically similar; Pancreatic leakage, operative time, blood loss: favor D2 lymphadenectomy.	Further rigorously controlled trials are required before conclusions on which procedure was more favorable could be made.

**Table 5:** Findings of meta-analyses: An overview of included studies regarding other topics of “Surgery”

First Author	Journal (Year)	Comparisons	OS	SR, DFS, RFS, PFS, Recurrence, TTF,TTP	Other	Major comments
**Palliative gastrectomy vs. non-palliative gastrectomy**						
Sun J[36]	BMC Cancer (2013)	Palliative gastrectomy vs. non-palliative gastrectomy.	Favor palliative gastrectomy (especially stage M1 GC)	NA.	NA.	Palliative gastrectomy for AGC might be associated with longer survival, especially for patients with stage M1 gastric cancer.
**Palliative gastrectomy with vs. without hepatic resection**						
Sun J[36]	BMC Cancer (2013)	(Included palliative gastrectomy with vs. without hepatic resection).	Favor gastrectomy+ hepatic resection.	NA.	NA.	Palliative gastrectomy combined with hepatic resection might improve survival.
**Drain vs. no drain after gastrectomy**						
Liu HP[44]	Dig Surg (2011)	Drain vs. no drain after gastrectomy.	NA.	NA.	Wound infection, postoperative pulmonary infection, intra-abdominal abscess, mortality, number of postoperative days until passing of flatus and initiation of soft diet: statistically similar. Postoperative complications, hospital stay: lower in the no-drain group.	Avoiding the use of abdominal drains might reduce drain-related complications and shorten hospital stay after gastrectomy.

**Table 6:** Findings of meta-analyses: An overview of included studies regarding chemotherapy vs. BSC

First Author	Journal (Year)	Comparisons	OS	RR	SR, DFS, RFS, PFS, Recurrence, TTF,TTP	Major comments
Badiani B[9]	World J Clin Oncol (2015)	6 regimens of chemotherapy vs. BSC	OS: paclitaxel with/without ramucirumab vs. BSC: favor chemotherapy; other 4 vs. BSC: statistically similar.	NA.	NA.	Both paclitaxel monotherapy and ramucirumab + paclitaxel determined a significant prolongation in survival as compared with BSC.
Iacovelli R[18]	PLoS One (2014)	(Include chemotherapy vs. BSC)	OS: patient ECOG=0: favor chemotherapy; patient ECOG≥1: favor chemotherapy.	NA.	NA.	Patients with symptomatic disease should not be immediately excluded by further lines of therapy.
Kim HS[31]	Ann Oncol (2013)	Second-line chemotherapy vs. BSC	Favor Second-line chemotherapy.	NA.	NA.	This study demonstrated evidence to support second-line chemotherapy in AGC.
Wagner AD[51]	Cochrane Database Syst Rev (2010)	(Include chemotherapy vs. BSC).	Favor chemotherapy	NA.	TTP: favor chemotherapy.	Chemotherapy significantly improved survival in comparison to BSC.
Casaretto L[60]	Braz J Med Biol Res (2006)	Chemotherapy vs. BSC	OS: 3-, 6-mo: statistically similar. 12-mo: favor chemotherapy.	Objective RR: statistically similar.	NA.	Chemotherapy increased the 1-y SR, provided a longer symptom-free period of 6 months and an improvement in quality of life.
Wagner AD[59]	J Clin Oncol (2006)	(Include chemotherapy vs. BSC).	Favor chemotherapy.	NA.	NA.	-

### Surgery

#### Laparoscopic gastrectomy (LG) *vs.* open gastrectomy (OG)

Nine meta-analyses compared the outcomes of LG *versus* OG [12, 13, 17, 26, 27, 29, 34, 38, 45]. There were 2, 5, and 3 meta-analyses comparing the 1-, 3-, 5-year survival, respectively [13, 17, 26, 27, 34]. Another two meta-analyses reported the overall survival (OS) without detailed time points [29, 45]. The meta-analyses all demonstrated that the OS was statistically similar between LG and OG [13, 17, 26, 27, 29, 34, 45]. Two of them did not report OS [12, 38]. As for disease-free survival (DFS), three meta-analyses compared the 3- and 5-year DFS [17, 26, 27] and another one reported DFS without detailed time point [29]. They all came to the conclusion that DFS was statistically similar between LG and OG [17, 26, 27, 29]. As for the recurrence, six of them found that it was statistically similar between the two groups [13, 17, 26, 27, 34, 38] while the other three did not report relevant data. Although LG required longer operative time [17, 26, 27, 34, 38, 45], it offered some advantages over OG with lower blood loss [13, 17, 26, 27, 34, 38, 45], shorter hospitalization [13, 17, 26, 27, 34, 38, 45] and quick recovery [13, 17, 26, 27, 38].

**Table 7:** Findings of meta-analyses: An overview of included studies regarding S-1-based therapy vs. 5-FU-based therapy

First Author	Journal (Year)	Comparisons	OS	RR	SR, DFS, RFS, PFS, Recurrence, TTF,TTP	Major comments
Ter Veer E[7]	Gastric Cancer (2016)	(Include S-1-based therapy vs. 5-FU- based therapy)	Statistically similar.	Objective RR: favor S-1-based therapy.	PFS: statistically similar.	S-1-based therapy showed no difference in survival, but the toxicity profile of S-1 was clearly more advantageous in Western patients.
Wu FL[14]	Medicine (Baltimore) (2015)	(Include S-1 based therapy vs. 5-FU- based therapy).	Favor S-1-based therapy.	Objective RR: favor S-1-based therapy.	PFS: favor S-1-based therapy.	S-1-based chemotherapy was favorable to AGC patients with better clinical benefit than 5-FU-based chemotherapy.
Yang J[24]	World J Gastroenterol (2014)	(Include S-1 based therapy vs. 5-FU -based therapy).	Favor S-1-based regimens.	Objective RR: favor S-1-based regimens.	PFS: statistically similar. TTF: favor S-1-based regimens	S-1-based chemotherapy prolonged OS and TTF, and induced less leukopenia and stomatitis.
Li DH[19]	Tumour Biol (2014)	S-1-based versus 5-FU-based chemotherapy	Favor S-1-based therapy	Overall RR: statistically similar.	TTF: statistically similar.	S-1-based therapy was favorable in OS and safety profile as first-line treatment in AGC. It was prone to improving overall RR and TTF, though the difference was not significant.
Liu H[21]	Medicine (Baltimore) (2014)	S-1+ paclitaxel vs. 5-FU+ paclitaxel	NA.	Overall RR: statistically similar.	Median PFS: favor S-1+ paclitaxel therapy. 6-mo PFS, TTF, TTP: statistically similar.	S-1+ paclitaxel therapy was a good alternative strategy for patients who could not tolerate a continuous intravenous infusion.
Huang J[42]	Med Oncol (2011)	S-1-based therapy vs. 5-FU-based therapy	Favor S-1-based therapy.	Overall RR: statistically similar.	NA.	S-1-based therapy significantly improved OS. Overall RR and safety profile were considerable between two groups.

**Table 8:** Findings of meta-analyses: An overview of included studies regarding S-1-based vs. capecitabine-based therapy

First Author	Journal (Year)	Comparisons	OS	RR	SR, DFS, RFS, PFS, Recurrence, TTF,TTP	Other	Major comments
Ter Veer E[7]	Gastric Cancer (2016)	(Include S-1-based vs. capecitabine-based therapy)	Statistically similar.	Objective RR: statistically similar.	PFS: statistically similar.	Grade 3-4 neutropenia and grade 1-2 hand-foot syndrome: lower in S-1-based therapy. Febrile neutropenia, serious AEs, toxicity-related deaths: statistically similar.	S-1-based therapy showed no difference in survival but showed a better toxicity profile compared with capecitabine based therapy.
Wu FL[14]	Medicine (Baltimore) (2015)	(Include S-1- based vs. capecitabine-based therapy)	Statistically similar.	Overall RR: statistically similar.	PFS: statistically similar.	Overall grade 3-4 toxicity: statistically similar. Grade 3 to 4 toxicity of diarrhea: lower in S-1-based therapy.	S-1-based chemotherapy had equivalent antitumor compared with capecitabine based therapy.
Yang J[24]	World J Gastroenterol (2014)	(Include S-1 based vs. capecitabine-based therapy).	Statistically similar.	Objective RR, TTF: statistically similar.	PFS: statistically similar.	Grade 3 or 4 AEs: statistically similar.	S-1 and capecitabine could be used for AGC interchangeably.
He MM[30]	PLoS One (2013)	S-1-based vs. capecitabine-based chemotherapy as first-line treatment.	OS, Survival probability (0.5-, 1-, 2-y): statistically similar.	Overall RR: statistically similar.	TTP: statistically similar. Progression-free probability: 3-mo, 6-mo: statistically similar.	Grade 3- 4 hematological and non-hematological toxicities: statistically similar (except hand-foot syndrome: less in S-1-based chemotherapy).	S-1-based chemotherapy was associated with non-inferior antitumor efficacy and better safety profile, We recommended S-1 and capecitabine could be used interchangeably for AGC, at least in Asia.

**Table 9:** Findings of meta-analyses: An overview of included studies regarding S-1-based and combination therapy

First Author	Journal (Year)	Comparisons	OS	RR	SR, DFS, RFS, PFS, Recurrence, TTF,TTP	Other	Major comments
**S-1 based vs. cisplatin based therapy**							
Wu FL[14]	Medicine (Baltimore) (2015)	(Include S-1 -based vs. cisplatin-based therapy)	Statistically similar.	Objective RR: statistically similar.	PFS: statistically similar.	NA.	-
**S-1 based combination therapy vs. S-1 monotherapy**							
Ter Veer E[7]	Gastric Cancer (2016)	(Include S-1-based combination therapy vs. S-1 monotherapy)	Favor S-1 combination therapy.	Objective RR: favor S-1 combination therapy.	PFS: favor S-1 combination therapy.	AEs: higher in S-1 combination therapy.	S-1 combination therapy was more efficacious than S-1 monotherapy.
Liu GF[20]	World J Gastroenterol (2014)	S-1-based combination therapy vs. S-1 monotherapy	Favor S-1 combination therapy.	Overall RR: favor S-1 combination therapy.	PFS: favor S-1 combination therapy.	Grade 3-4 leucopenia, neutropenia, diarrhea: higher in S-1 combination therapy.	S-1-based combination therapy was superior to monotherapy in terms of OS, PFS and overall RR.
Wu JR[23]	Tumour Biol (2014)	S-1-based combination therapy vs. S-1 monotherapy.	Favor S-1 combination therapy.	Objective RR: favor S-1 combination therapy.	PFS: favor S-1 combination therapy.	Grade 3/4 toxicity event: higher in S-1 combination therapy.	For the Asian population, S-1 combination therapy improved OS and PFS and enhanced objective RR. The safety profile was poorer in patients with S-1 combination therapy

**Table 10:** Findings of meta-analyses: An overview of included studies regarding oxaliplatin-based vs. cisplatin-based therapy

First Author	Journal (Year)	Comparisons	OS	RR	SR, DFS, RFS, PFS, Recurrence, TTF,TTP	Other	Major comments
Montagnani F[46]	Gastric Cancer (2011)	Oxaliplatin vs. cisplatin.	NA.	NA.	PFS: favor oxaliplatin.	Neutropenia, thromboembolic: lower in oxaliplation. Neurotoxicity: higher in oxaliplation.	A small but significant survival benefit of oxaliplatin was associated with less toxicity and better tolerability, especially in older patients and when used in two-drug, bi-weekly regimens.
Wagner AD[51]	Cochrane Database Syst Rev (2010)	(Include oxaliplatin-vs. cisplatin-containing regimens).	Statistically similar	Objective RR: favor oxaliplatin -based therapy.	PFS:statistically similar.	Treatment related death, treatment discontinuation due to toxicity: statistically similar.	These results confirmed the non-inferiority of oxaliplatin, as compared to cisplatin, in the treatment of AGC.
Gong JF[55]	Zhonghua Yi Xue Za Zhi (2009)	Oxaliplatin- based chemotherapy vs. cisplatin -based chemotherapy	OS: 1-y: favor oxaliplatin-based chemotherapy	Objective RR: favor oxaliplatin-based chemotherapy.	NA.	Peripheral neurotoxicity: higher in oxaliplatin-based chemotherapy; Anemia/nusea/vomiting: higher in cisplatin-based chemotherapy.	Oxaliplatin-based chemotherapy was well-tolerated and more effective than cisplatin in AGC.

**Table 11:** Findings of meta-analyses: An overview of included studies regarding capecitabine-based therapy vs.5-FU -based therapy

First Author	Journal (Year)	Comparisons	OS	RR	SR, DFS, RFS, PFS, Recurrence, TTF,TTP	Other	Major comments
Xu HB[15]	Eur J Clin Pharmacol (2015)	Capecitabine+ oxaliplatin (XELOX) vs. 5-fluorouracil/ leucovorin+ oxaliplatin (FOLFOXs)	NA.	Overall RR: statistically similar	NA.	Clinical benefit rate: statistically similar. Nausea, stomatitis, diarrhea and alopecia: lower in capecitabine regimen. Hand-foot syndrome: higher in capecitabine based regimen.	Owing to limited data and potential bias of the included studies, further rigorously controlled trials are required.
Ma Y[40]	J Clin Pharm Ther (2012)	Capecitabine-based vs.5-FU-based therapy.	Favor capecitabine-based chemotherapy	Overall RR: favor capecitabine-based chemotherapy	NA.	Grade 3 or 4 leukopenia, stomatitis and nausea and vomiting, hand-foot syndrome: lower in capecitabine-based regimens; Haematological toxicity: statistically similar	Capecitabine based chemotherapy strategies showed prolonged OS and enhanced overall RR.AGC. Asian patients also showed less grade 3/4 gastrointestinal toxicity with the capecitabine based regimens compared with Caucascian patients.
Wagner AD[51]	Cochrane Database Syst Rev (2010)	(Include regimens containing oral 5-FU prodrugs vs. intravenous fluoropyrimidines).	Statistically similar	Objective RR: favor capecitabine- containing regimen	PFS: statistically similar.	Treatment related death, treatment discontinuation due to toxicity: statistically similar.	Gastric cancer patients with adequate renal function and compliance should be treated with capecitabine instead of 5-FU.

**Table 12:** Findings of meta-analyses: An overview of included studies regarding CPT-11-based therapy vs. non CPT-11-based therapy

First Author	Journal (Year)	Comparisons	OS	RR	SR, DFS, RFS, PFS, Recurrence, TTF,TTP	Other	Major comments
Qi WX[35]	Int J Cancer (2013)	CPT-11-containing vs. non-CPT-11-containing regimen.	Favor CPT-11-containing regiments.	Overall RR: statistically similar.	1-y SR, TTF: statistically similar; PFS: favor CPT-11-containing regiments.	Grade 3/4 fatigue: higher in CPT-11-containing regimen.	The study provided strong evidence for a survival benefit of CPT-11-containing regimen as first-line treatment for AGC.
Wang DL[52]	World J Gastroenterol (2010)	CPT-11-based chemotherapy vs. non CPT-11-based chemotherapy.	Statistically similar	Overall RR: statistically similar.	TTF: favor CPT-11-containing chemotherapy	Grade 3/4 haemotological toxicity and gastrointestinal toxicity: lower in CPT-11- containing	CPT-11-based therapy was advantageous over non CPT-11-based chemotherapy for TTF with no significant toxicity.
Wagner AD[51]	Cochrane Database Syst Rev (2010)	(Include CPT-11-containing vs. non-CPT-11-containing regimen).	Statistically similar	Objective RR: statistically similar.	PFS: statistically similar.	Treatment related death, treatment discontinuation due to toxicity: statistically similar.	CPT-11-containing regimens should be considered as a true and at least equally effective alternative to platinum-based combinations in first-line therapy
Wagner AD[59]	J Clin Oncol (2006)	(Include CPT-11- containing vs. non CPT-11- containing therapy).	Statistically similar	NA.	NA.	Treatment related death: statistically similar.	CPT-11-containing regimens exhibited a benefit in survival and a lower rate of treatment-related deaths although these differences were statistically non-significant.

**Table 13:** Findings of meta-analyses: An overview of included studies regarding platinum-based and vs. cisplatin -based therapy

First Author	Journal (Year)	Comparisons	OS	RR	SR, DFS, RFS, PFS, Recurrence, TTF,TTP	Other	Major comments
**Platinum based vs. non-platinum based therapy**							
Chen WW[28]	PLoS One (2013)	Platinum-based vs. non platinum -based therapy	Platinum-based vs. old-generation agents: favor platinum-based therapy; Platinum-based vs. g new-generation agents: statistically similar.	RR: platinum-based vs. old-generation agents: favor platinum-based therapy; Platinum-based vs. g new-generation agents: statistically similar.	NA.	Hematological toxicity, non-hematological toxicity: higher in platinum-based regimens. Thrombocytopenia (only in platinum-based therapy vs. old-generation agents), nephrotoxicity (only in platinum-based therapy vs. new-generation agents), toxic death rate: statistically similar.	New-generation agent (S-1, taxanes and irinotecan) based combination regimens achieved similar RR and OS as platinum-based therapy that had generally higher side effects. S-1, taxanes and irinotecan seemed to be valid options for patients with inoperable, advanced gastric cancer as first-line chemotherapy.
**Cisplatin vs. non cisplatin chemotherapy**							
Petrelli F[33]	PLoS One (2013)	Chemotherapy with vs. without cisplatin	Favor therapy without cisplatin.	RR: favor therapy without cisplatin	PFS: favor therapy without cisplatin	NA.	New active cytotoxic agents instead of cisplatin significantly enhanced OS, PFS, and RR in first-line treatment of metastatic GC.

**Table 14:** Findings of meta-analyses: An overview of included studies regarding targeted chemotherapy

First Author	Journal (Year)	Comparisons	OS	RR	SR, DFS, RFS, PFS, Recurrence, TTF,TTP	Other	Major comments
Ciliberto D[10]	Cancer Biol Ther (2015)	Targeted therapy vs. conventional therapy	Favor targeted therapy of anti-angiogenic and HER2 but not EGFR pathway	RR: favor anti-HER2 agents but not for anti-EGFR and anti-angiogenic agents.	PFS: favor targeted therapy of anti-angiogenic and HER2 but not EGFR pathway.	Diarrhea: higher in anti-HER2 agents. Rash: higher in anti-EGFR drugs.	Targeted therapy showed a significant survival benefit, which can be ascribed to anti-angiogenic and anti-HER2 agents.
Iacovelli R[18]	PLoS One (2014)	Targeted therapy vs. BSC or traditional chemotherapy	OS: patients ECOG=0: statistically similar; targeted therapy vs. chemotherapy: favor chemotherapy. In patients with ECOG≥1: targeted therapy vs. BSC: favor targeted therapy; targeted therapy vs. chemotherapy: statistically similar;	NA.	NA.	NA.	In patients with ECOG-PS = 0, ramucirumab and everolimus did not report a significant survival benefit. Any active therapy over BSC was more effective on patients with ECOG-PS = 1 or more. Patients with symptomatic disease should not be immediately excluded by further lines of therapy.
Qi WX[22]	Tumour Biol (2014)	Anti-VEGF agents vs. non anti-VEGF agents	Favor anti-VEGF therapy	Objective RR: favor anti-VEGF therapy	RFS: favor anti-VEGF therapy	Grade 3 or 4 thrombocytopenia, diarrhea, and hypertension: higher in anti-VEGF therapy	The anti-VEGF therapy offered a significant survival benefit in patients with AGC, especially for those previously treated patients.

**Table 15:** Findings of meta-analyses: An overview of included studies regarding combination (doublet/triplet) therapy vs. single/doublet therapy

First Author	Journal (Year)	Comparisons	OS	RR	SR, DFS, RFS, PFS, Recurrence, TTF,TTP	Other	Major comments
Zhang Y[8]	Medicine (Baltimore) (2016)	Doublet combination therapy vs. single theapy	Favor doublet combination therapy (targeted agent+cytotoxic chemotherapy improved OS, but not for doublet cytotoxic chemotherapy)	Objective RR: favor doublet combination therapy	PFS: favor doublet combination therapy (also significant in targeted agent+ chemotherapy compared wth single cytotoxic agent).	Grade 3/4 myelosuppression, toxicities, diarrhea, fatigue: higher in doublet combination therapy; Grade 3/4 thrombocytopenia, nausea: statistically similar.	The addition of targeted agent to mono-chemotherapy as salvage treatment for pretreated AGC patients provided substantial survival benefits, while no significant survival benefits were observed in doublet cytotoxic chemotherapy regimens.
Liu N[39]	Chin J Hos Pharm (2012)	Triplet chemotherapy vs. doublet chemotherapy	NA.	Overall RR: favor triplet combination chemotherapy.	NA.	Effectiveness: favor triplet combination chemotherapy; Grade 3/4 AEs: statistically similar;	Triplet chemotherapy was more effective for AGC patients compared to double chemotherapy, esp taxoid based triplet chemotherapy.
Wagner AD[51]	Cochrane Database Syst Rev (2010)	(Include combination vs. monotherapy)	Favor combination chemotherapy.	Objective RR: favor combination chemotherapy.	TTP: favor combination chemotherapy.	Toxicity: higher in combination chemotherapy.	Combination chemotherapy improved survival compared to single-agent therapy.
Wagner AD[59]	J Clin Oncol (2006)	(Include combination vs. monotherapy)	Favor combination chemotherapy.	NA.	NA.	Toxicity: higher in combination therapy. Treatment related death: statistically similar.	Combination chemotherapy improved survival compared to single-agent therapy.

**Table 16:** Findings of meta-analyses: An overview of included studies regarding FU/anthracycline/cisplatin combination therapy

First Author	Journal (Year)	Comparisons	OS	RR	SR, DFS, RFS, PFS, Recurrence, TTF,TTP	Other	Major comments
**FU/anthracycline-containing combinations with vs. without cisplatin**							
Wagner AD[51]	Cochrane Database Syst Rev (2010)	(Include 5-FU/ anthracycline-containing combination therapy with vs. without cisplatin).	Favor three-drug combination.	NA.	NA.	NA.	The comparisons confirmed a statistically significant advantage in overall survival for the three-drug combination though this benefit was achieved at the price of significant toxicity
Wagner AD[59]	J Clin Oncol (2006)	(Include FU/ anthracycline-containing combination therapy with vs. without cisplatin).	Favor three-drug combination.	NA.	NA.	NA.	Best survival results were achieved with three-drug regimens containing FU, an anthracycline, and cisplatin.
**FU/cisplatin-containing regimens with vs. without anthracyclines**							
Wagner AD[51]	Cochrane Database Syst Rev (2010)	(Include 5-FU/cisplatin-containing combination therapy regimens with vs. without anthracyclines).	Favor three-drug combination.	NA.	NA.	NA.	The comparisons confirmed a statistically significant advantage in overall survival for the three-drug combination though this benefit was achieved at the price of significant toxicity
Wagner AD[59]	J Clin Oncol (2006)	(Include FU/cisplatin- containing regimens with vs. without anthracyclines).	Favor three-drug combination.	NA.	NA.	NA.	Best survival results were achieved with three-drug regimens containing FU, an anthracycline, and cisplatin.

**Table 17:** Findings of meta-analyses: An overview of included studies regarding docetaxel, lentinan and postoperative intravenous chemotherapy

First Author	Journal (Year)	Comparisons	OS	RR	SR, DFS, RFS, PFS, Recurrence, TTF,TTP	Other	Major comments
**Docetaxel versus non docetaxel-containing regimens**							
Wagner AD[51]	Cochrane Database Syst Rev (2010)	(Include docetaxel-containing vs. non docetaxel- containing regimens).	Statistically similar	Objective RR: statistically similar	TTP: statistically similar.	Treatment related death, treatment discontinuation due to toxicity: statistically similar.	The clinical value of docetaxel-containing regimen was regarded as controversial.
**Chemotherapy regimens with vs. without lentinan administration**							
Oba K[56]	Anticancer Res (2009)	Chemotherapy regimens with vs. without lentinan administration	Favor with lentinan combination therapy.	NA.	NA.	NA.	The addition of lentinan to standard chemotherapy offered a significant advantage over chemotherapy alone in terms of survival.
**EPIPC vs. early postoperative intravenous chemotherapy**							
Zhang YL[54]	Chin Gen Prac (2011)	EPIPC vs. early postoperative intravenous chemotherapy	NA.	NA.	SR: 1-, 2-, 3-, 5- y: favor EPIPC. Replase rate: 2-, 3-y intra-abdominal recurrence: favor EPIPC.	Nausea, vomiting: lower in EPIPC. Liver and renal function protection: favor EPIPC	EPIPC improved survival rate and reduced both recurrence rate and side effects.

**Table 18:** Findings of meta-analyses: An overview of included studies regarding TCM

First Author	Journal (Year)	Comparisons	OS	RR	Other	Major comments
**SQFZ injection + chemotherapy vs. chemotherapy alone**						
Li J[11]	Chin J Integr Med (2015)	SQFZ injection+ chemotherapy vs. chemotherapy alone	NA.	NA.	Quality of life, complete remission and partial remission, AEs: favor SQFZ injection+ chemotherapy.	This systematic review found encouraging albeit limited evidence for SFI combined with chemotherapy.
Yao K[25]	J Cancer Res Ther (2014)	SQFZ injection + chemotherapy vs. chemotherapy alone	NA.	Overall RR: favor SQFZ +chemotherapy.	The Karnofsky score (KPS): higher in SQFZ injection+ chemotherapy.	SQFZ+ chemotherapy could improve the clinical efficacy and performance status in patients with AGC.
**Huachansu+chemotherapy vs. chemotherapy alone**						
Xie X[37]	Med Hypotheses (2013)	Huachansu+ chemotherapy vs. chemotherapy alone	OS: 1-year: statistically similar.	NA.	Total RR, KPS, gastrointestinal side effects, leucocytopenia: favor Huachansu+ chemotherapy	Huchansu+ chemotherapy improved RR, increased Karnofsky score and reduced leucocytopenia.
**Compound matrine injection+cisplatin chemotherapy vs. cisplatin chemotherapy**						
Huang S[43]	China J Chin Mat Med (2011)	Compound matrine injection+ cisplatin vs. cisplatin	NA.	NA.	Quality of life, clinical efficacy, leukopenia, thrombocytopenia, gastrointestinal AEs: favor combination therapy.	Compound matrine injection+ cisplatin chemotherapy could improve the quality of life with lower AEs.
**KLT+chemotherapy vs. chemotherapy alone**						
Wang C[47]	Mod J Integr Tradi Chin West Med (2011)	KLT+ chemotherapy vs. chemotherapy alone	OS: 1-y: KLT+ chemotherapy.	NA.	Quality of life, clinical efficacy, liver function, cachexia, AEs: favor KLT+ chemotherapy.	KLT+ chemotherapy improved the curative effect and survival rate with lower incidence of AEs.

*no data was reported regarding SR, DFS, RFS, PFS, Recurrence, TTF,TTP in the included studies

Besides, three of them demonstrated that LG had fewer postoperative complications [13, 17, 26], but another two did not show significant difference between the two groups [34, 38]. In addition, LG was comparable to OG that the number of harvested lymph nodes was statistically similar between two groups [12, 13, 17, 26, 27, 34, 38, 45].

Only non-randomized controlled trial (RCT) studies were included in the meta-analyses by Lu (*n* = 8) and Qiu (n = 7). The meta-analyses by Huang did not report type of the included studies. In the meta-analyses by Martinez-Ramos, Choi, Ye, Zou and Quan, there was only one RCT regarding LG *versus* OG while Chen's meta-analysis had a larger number of RCTs.

The meta-analyses by Quan had the largest number of included studies (*n* = 26) followed by the meta-analyses by Chen (*n* = 15) and Zou (*n* = 14) (Supplementary Table S5). By comparison, the number of included studies was less than 20 in 8 other meta-analyses.

Given its superiority in the quantity of RCT studies, the results of the meta-analysis by Chen might be more reliable. In details, LG was a safe and technical alternative to OG for AGC with a lower complication rate and enhanced postoperative recovery.

#### Neoadjuvant chemotherapy (NAC) *vs.* no therapy before surgery

Two meta-analyses compared the outcomes of surgery in combination with NAC *versus* no therapy before surgery. Both of them favored NAC in terms of OS [41, 50]. One of them also favored NAC in terms of 3-year progression-free survival (PFS) [50] while the other did not reported PFS. Besides, both of the meta-analyses demonstrated that the resection rate was higher for NAC group than for control group while the perioperative mortality showed no statistically difference [41, 50]. Additionally, one of them revealed that NAC had a significant down-staging effect on AGC [50].

Only RCT studies were included in the meta-analysis by Xiao (*n* = 18) and the meta-analysis by Li (*n* = 14) did not report the details of the included studies.

The meta-analysis by Xiao had a larger number of included studies than those by Li (18 *versus* 14) (Supplementary Table S6). Notably, there was an overlap of included studies between the two meta-analyses. All studies that were included in the meta-analysis by Li were also covered by the meta-analysis by Xiao.

Given its superiority in the quantity of RCT studies, the results of the meta-analysis by Xiao might be reliable. In details, NAC could improve the tumor resection rate and the survival rate in AGC patients without increasing the operative risk. Besides, perioperative mortality in two groups was statistically similar.

#### Surgery with *vs.* without AC

Two meta-analyses compared the outcomes of surgery combined with AC *versus* surgery without AC. Both of them favored surgery combine with AC. Other relevant outcomes were not evaluated in both meta-analyses [36, 57].

Only RCT studies were included in meta-analysis by Sun P while only non RCTs were included in Sun J's article.

The meta-analysis by Sun P had the larger number of included studies (*n* = 12) compared to the meta-analysis by Sun J (*n* = 3) (Supplementary Table S7). Notably, there was an overlap of included studies between them.

Given its superiority in the quantity of RCT studies, the results of the meta-analysis by Sun P might be more reliable. In details, postoperative chemotherapy could improve OS after radical surgery for gastric cancer.

#### Surgery with *vs*. without intraperitoneal chemotherapy (IPC)

Two meta-analyses compared the outcomes of surgery combined with IPC *versus* surgery without IPC [16, 58]. One of the meta-analyses revealed IPC significantly improve 1-, 2-, 3-year OS but not 5-year OS [16]. Another meta-analysis which also focused on adjuvant IPC demonstrated that hyperthermic intraoperative intraperitoneal chemotherapy (HIIPC) with or without early postoperative intraperitoneal chemotherapy (EPIPC) after gastrectomy was associated with improved OS; however, OS was statistically similar between surgery with normothermic intraoperative intraperitoneal chemotherapy (NIIPC), EPIC or delayed postoperative intraperitoneal chemotherapy (DPIPC) and surgery without IPC [58].

As for recurrence, one indicated that the overall recurrence and the peritoneal recurrence rates were improved by surgery combined with IPC [16]. However, another meta-analysis demonstrated that the peritoneal recurrence rates did not improved by surgery combined with IPC [58]. Besides, there was no significant difference in lymph-nodal recurrence rate; and the rate of haematogenous metastasis was improved by surgery combined with IPC [16].

As for mortality, one meta-analysis revealed that 1-, 2- and 3-year overall mortality was improved by IPC while 5-year overall mortality had no statistically difference between the two groups. Besides, 1- and 2-year mortality rate in patients with serosal infiltration as well as 2- and 3-year mortality rate in patients with loco-regional nodal metastasis were improved by the use of IPC. But morbidity rate is incremented by IPC [16]. Another meta-analysis showed that IPC did not improve the perioperative mortality compared to surgery alone. Besides, risk of intra-abdominal abscess and neutropenia were increased by IPC [58].

Only RCT studies, rather than non-RCTs, were included in the two meta-analyses.

The meta-analysis by Coccolini had the largest number of included studies (*n* = 20) followed by the meta-analysis by Yan (*n* = 13) (Supplementary Table S8). Notably, there was an overlap of included studies among them.

Given its superiority in the quantity of RCT studies, the results of the meta-analysis by Coccolini might be more reliable. In details, IPC had positive effect on overall and peritoneal recurrence and distant metastasis. Besides, loco-regional lymph-nodes invasion in patients affected by AGC was not a contraindication to IPC.

#### D2 *vs*. D4 lymphadenectomy

Two meta-analyses compared D2 lymphadenectomy *versus* D2 + para-aortic nodal dissection, which is also called D4 lymphadenectomy. Both of them demonstrated that there were no significant differences between two groups in 5-year OS or 5-year survival rate [53, 54]. One of them showed that the 5-year recurrence was also statistically similar between two groups [54] while the other did not report relevant data.

Both RCT and non-RCT studies were included in the meta-analysis by Wang and all studies included in Zhang's article were RCT studies.

The meta-analysis by Wang had a larger number of included studies than those by Zhang (11 *versus* 4) (Supplementary Table S9). Notably, there was an overlap of included studies between the two meta-analyses by Wang and Zhang. All studies that were included in the meta-analysis by Zhang were also covered by the meta-analysis by Wang.

Given its superiority in the quantity of RCT studies, the results of the meta-analysis by Wang might be reliable. In details, D2 plus PAND (D4) could be performed as safely as standard D2 resection without increasing postoperative mortality but failed to benefit OS in patients with AGC. However, D2 group was favored in terms of operation time and blood loss.

#### D2 *vs*. D3 lymphadenectomy

One meta-analysis compared the outcomes of D2 *versus* D3 lymphadenectomy. There was no significant difference between two groups regarding 5-year survival rate. So were postoperative mortality, anastomotic leakage, and pneumonia. Additionally, D2 dissection was favorable in terms of operation time, blood loss during surgery and postoperative pancreatic leakage. Owing to the limitation in statistically methods of the included studies, further rigorously controlled trials are required before conclusions on which procedure was more favorable could be made [48].

#### Palliative gastrectomy *vs*. non-palliative gastrectomy

One meta-analysis compared the outcomes of palliative gastrectomy *versus* non-palliative gastrectomy. Palliative gastrectomy for patients with incurable AGC was associated with longer OS, especially for patients with stage M1 GC [36].

#### Palliative gastrectomy with *vs*. without hepatic resection

One meta-analysis compared the outcomes of palliative gastrectomy combined with hepatic resection *versus* gastrectomy without hepatic resection in AGC patients with liver metastasis. The meta-analysis confirmed that palliative gastrectomy combined with hepatectomy might provide better OS than palliative gastrectomy only [36].

#### Drain *vs*. no-drain after gastrectomy

One meta-analysis evaluated drain *versus* no-drain after gastrectomy. The study did not report survival data. There were no significant differences between the drain and no-drain groups in terms of wound infection, postoperative pulmonary infection, intra-abdominal abscess, mortality, number of postoperative days until passing of flatus and initiation of soft diet. Besides, the results favored no drain after gastrectomy regarding postoperative complications and the length of hospital stay [44].

### Chemotherapy

#### Chemotherapy *vs*. basic supportive care (BSC)

Six meta-analyses compared the outcomes of chemotherapy with BSC [9, 18, 31, 51, 59, 60]. Five of them reported OS without detailed time points showing that OS was improved by chemotherapy [9, 18, 31, 51, 59]. Among them, one meta-analysis comparing six regimens of chemotherapy with BSC demonstrated that OS was only favorable in paclitaxel and ramucirumab + paclitaxel *vs*. BSC while the other four chemotherapy regimens showed no statistical difference [9]. Another meta-analysis revealed that the 3- and 6- month OS was statistically similar between chemotherapy and BSC. However, chemotherapy was favorable in terms of 12-month OS [60]. One meta-analysis reported response data, which indicated that the objective response rate (RR) was statistically similar between the two groups [60]. Beside, one of them favored chemotherapy regarding time to progression (TTP) [51]. Chemotherapy brought benefit in terms of symptom-free period, quality of life and tumor mass reduction according to the results of one of the meta-analyses [60].

Only RCT studies, rather than non-RCTs were included in the six meta-analyses. The meta-analysis by Badiani had the largest number of included studies (*n* = 7), followed by the meta-analysis by Casaretto (*n* = 5) (Supplementary Table S10). Notably, there was an overlap of included studies between the two meta-analyses by Wagner AD published in 2010 and 2006, respectively. All related studies included in the meta-analysis by Wagner in 2010 were also covered by the one he wrote in 2006.

Given its superiority in the quantity of RCT studies, the results of the study by Badiani might be more reliable. In details, both paclitaxel monotherapy and ramucirumab plus paclitaxel therapy determined a significant prolongation in survival as compared with BSC.

#### S-1-based therapy *vs*. 5-FU-based therapy

Six meta-analyses compared S-1-based therapy with 5-FU-based therapy [7, 14, 19, 21, 24, 42]. Five of them compared OS [7, 14, 19, 24, 42]. Among them, four of them favored S-1-based therapy [14, 19, 24, 42] while one meta-analysis showed that there was no statistically difference between two groups in terms of OS [7]. As for objective RR or overall RR, three of them favored S-1 in terms of objective RR [7, 14, 24] while the other three meta-analyses suggested that overall RR was statistically similar between the two groups [19, 21, 42]. Four meta-analyses reported PFS [7, 14, 21, 24,]. Two of them showed that the PFS was statistically similar between the S-1-based and 5-FU-based therapy [7, 24]; one favored S-1-based therapy over 5-FU-based therapy [14]; the other one revealed that S-1-based therapy was favorable in terms of median PFS but the 6-month PFS was statistically similar between two groups [21]. Three of them compared time to failure (TTF) [19, 21, 24]; one favored S-1-based regimen [24] while the other two showed that there were no significant differences between two groups [19, 21]. Only one meta-analysis reported TTP that it was statistically similar between two groups when it comes to TTP [21]. According to one of the included meta-analyses, S-1-based therapy was favorable regarding the incidence of progressive disease and disease control rate while the incidence of stable disease was significant similar between two groups [21]. One meta-analysis evaluated drug-related toxicity based on sub-analysis. In the Western subgroup, S-1-based therapy demonstrated significantly lower rates of febrile neutropenia, toxicity-related deaths, grade 3-4 stomatitis and mucositis and grade 1-2 diarrhea, stomatitis and alopecia compared with 5-FU-based therapy; besides, the rates of grade 1-2 neutropenia and hand-foot syndrome were greater with S-1 than with 5-FU. In the Asian subgroup, S-1-based therapy showed a significantly increased incidence of grade 3-4 fatigue and grade 1-2 abdominal pain but a lower incidence of grade 1-2 neutropenia, nausea and weight loss compared with 5-FU-based therapy [7]. The incidence of febrile neutropenia, serious adverse event (AEs)or toxicity-related deaths was statistically similar between both arms [7]. One meta-analysis favored S-1 therapy regarding grade 3/4 nausea [14] but another one meta-analysis showed that there was no significant difference between two groups [42]. Three meta-analyses reported stomatitis that two of them favored S-1-based therapy [19, 24] while the other one revealed no statistically difference between two groups [42]. Two meta-analyses reported grade 3/4 neutropenia. Both of them favored S-1-based therapy [14, 42]. One meta-analysis compared overall grade 3/4 toxicity that there was no significant difference between S-1-based and 5-FU-based therapy [14].

Only RCT studies, rather than non-RCTs, were included in the six meta-analyses.

The meta-analysis by Ter had the largest number of included studies, followed by the meta-analyses by Wu and Li (8 *versus* 6 *versus* 6) (Supplementary Table S11). Notably, there was an overlap of included studies between the three meta-analyses by Wu, Li and Ter. The included studies were completely same between the two meta-analyses by Wu and Li, which were also covered by the meta-analysis by Ter.

Given its superiority in the quantity of RCT studies, the results of the meta-analysis by Ter might be reliable. In details, S-1-based therapy showed no difference in survival compared with 5-FU-based therapy, but the toxicity profile of S-1 was clearly more advantageous in Western patients. In addition, the two groups were statistically similar in terms of febrile neutropenia, serious AEs, toxicity-related death (Asian).

#### S-1-based therapy *vs*. capecitabine-based therapy

Four meta-analyses compared the outcomes of S-1-based therapy *vs*. capecitabine-based therapy [7, 14, 24, 30]. As for OS, all the included meta-analyses demonstrated that the survival was statistically similar between the two groups. Besides, one meta-analysis that reported detailed survival data showed that the 0.5-, 1-, and 2-year survival probability was statistically similar between the two groups [30]. As for objective RR and overall RR, three of them found that that the two chemotherapy had statistically similar efficacy in terms of objective RR [7, 14, 24]; another meta-analysis that compared overall RR showed that there was no significant difference between two groups [30]. There were three meta-analyses to compare PFS. All of them demonstrated that PFS was statistically similar between two groups [7, 14, 24]. Additionally, another meta-analysis evaluated TTP and progression-free probability showed that TTP, 3- and 6-month progression-free probability was statistically similar between two regimens [30]. All the meta-analyses found that most of the grade 3-4 toxicity or adverse events AEs were statistically similar between two groups. When it comes to grade 1-2 hand-foot syndrome, one of them indicated that S-1-based therapy was favorable over capecitabine-based therapy [7].

Both RCTs and non-RCTs were included in the meta-analyses by Wu and He while only RCTs were included in the meta-analyses by Ter and Yang.

The meta-analysis by He had the largest number of included studies (*n* = 10) followed by the meta-analyses by Wu (*n* = 8), Ter (*n* = 3) and Yang (*n* = 2) (Supplementary Table S12). All studies that were included in the meta-analysis by Yang (*n* = 2) were also covered in the other three meta-analyses. Besides, all studies included in the meta-analysis by Wu were also covered by the article of He.

Given its superiority in the quantity of RCT studies, the results of the meta-analyses by He and Wu might be reliable. The results of them were consistent with each other. In details, S-1-based chemotherapy was associated with non-inferior antitumor efficacy and better safety profile, compared with capecitabine-based therapy. It was recommended that S-1 and capecitabine could be used interchangeably for AGC, at least in Asia. Besides, Grade 3 to 4 hematological toxicities between two groups were statistically similar except hand-foot syndrome, which was less in S-1-based therapy.

#### S-1-based therapy *vs*. cisplatin-based therapy

The efficacy of S-1-based and cisplatin-based therapy were evaluated in one meta-analysis. OS, objective RR and PFS were statistically similar between two groups. The meta-analysis did not report other survival data [14].

#### S-1-based combination therapy *vs*. S-1 monotherapy

Three meta-analyses compared the outcomes of S-1- based combination and momotherapy. All the included meta-analyses indicated that S-1 combination therapy was superior to monotherapy in terms of OS and PFS [7, 20, 23]. As for RR, two of them favored S-1 combination therapy in objective RR [7, 23] while another one meta-analysis indicated that S-1 was favorable regarding overall RR [20]. All the meta-analyses demonstrated that S-1 combination therapy was associated with higher incidence of AEs or grade 3-4 toxicity event [7, 20, 23].

Only RCTs studies, rather than non-RCTs were included in the meta-analyses by Ter and Liu. Both RCTs and non-RCTs were included in Wu's meta-analysis.

The meta-analysis by Ter had the largest number of included studies (*n* = 8) followed by the meta-analyses by Wu (*n* = 6) and Liu (*n* = 4) (Supplementary Table S13). Notably, there was an overlap of included studies between the three meta-analyses. All studies that were included in the meta-analysis by Liu were covered in Ter's and Wu's meta-analyses.

Given its superiority in the quantity of RCT studies, the results of the meta-analysis by Ter might be reliable. In details, S-1 combination therapy was more efficacious than S-1 monotherapy. However, AEs was higher and safety profile was poorer in S-1 combination therapy. This result was also confirmed by the other two meta-analyses.

#### Oxaliplatin-based therapy *vs*. cisplatin-based therapy

There were three meta-analyses to compare the outcomes of oxalipatin-based therapy *versus* cisplatin -based therapy [46, 51, 55]. Two of them evaluated OS. One found that survival was statistically similar between two groups [51]. But the other one favored oxalipatin- based therapy in terms of 1-year OS [55]. Two of them indicated that oxaliplatin-based therapy was superior to cisplatin-based therapy in terms of objective RR [51, 55] while one meta-analysis did not report RR data [46]. PFS was evaluated in two meta-analyses. One favored oxaliplatin while the other one showed no significant difference between two regimens. Oxaliplatin-based therapy was found to have higher neurotoxicity [46, 55] while cisplatin-based therapy had higher risk of neutropenia and thromboembolic events as well as anemia, nausea and vomiting events [55]. One meta-analysis revealed that the treatment related death and treatment discontinuation due to toxicity was statistically similar between two groups [51].

Only RCTs were included in two of the included meta-analyses except the one by Gong, which was not mentioned in the article.

The meta-analysis by Gong had the largest number of included studies (*n* = 16) (Supplementary Table S14). By comparison, the number of included studies was less than 10 in 2 other meta-analyses. Notably, there was an overlap of included studies between the meta-analyses by Wagner and Montagnani. All studies that were included in the meta-analysis by Wagner were also covered in the meta-analysis by Montagnani.

Given its superiority in the quantity of RCT studies, the results of the meta-analysis by Montagnani might be more reliable. In details, oxaliplatin based therapy was associated with a small but significant survival benefit with less toxicity and better tolerability, especially in older patients and when used in two-drug, biweekly regimens.

#### Capecitabine-based Capecitabine based therapy *vs*. 5-FU-based therapy

Three meta-analyses compared the outcomes of capecitabine-based therapy *versus* 5-FU-based therapy [15, 40, 51]. Capecitabine-based therapy was superior to 5-FU-based therapy in terms of OS according to one of the meta-analyses [40] while another meta-analysis showed that the survival was statistically between two groups [51]. And the other one did not report the survival data [15]. Besides, two of them found that capecitabine based therapy was associated with higher overall RR compared to 5-FU based therapy [40, 51]. On the contrary, the other one showed no significant difference between two groups [15]. PFS was evaluated in only one meta-analysis, which indicated that PFS was statistically similar between two groups. Two of meta-analyses showed that incidence of nausea and stomatitis was lower in capecitabine based therapy [15, 40]. Higher frequency of hand-foot syndrome was observed in capecitabine-based therapy according to one of the meta-analyses [15] while the other one showed the opposite results [40]. And the other meta-analysis suggested that treatment related death as well as treatment discontinuation due to toxicity was statistically similar between two regimens [51].

Only RCTs, rather than non-RCTs were included in the three meta-analysis.

The meta-analyses by Xu had the largest number of included studies (*n* = 26) followed by the meta-analyses by Ma (*n* = 18) and by Wagner AD (*n* = 1) (Supplementary Table S15). Notably, there was an overlap of included studies between them.

Given its superiority in the quantity of RCTs studies, the results of the meta-analysis by Xu might be more reliable. In details, the evidence showed that XELOX (capecitabine-based therapy) might share similar efficacy as FOLFOXs (5-FU-based therapy) and reduced toxicities of chemotherapy in AGC therapy. However, owing to limited data and potential bias of the included studies, confirmation of these conclusions in rigorously controlled, randomized trials is required before more firm conclusions about this therapy can be drawn.

#### Irinotecan (CPT-11)-based therapy *vs*. non CPT-11 -based

The efficacy of CPT-11-based therapy *versus* non-CPT-11-based therapy was summarized in four meta-analyses [35, 51, 52, 59]. When it comes to OS, three of them demonstrated that the survival was statistically similar [51, 52, 59] while one meta-analysis favored CPT-11-containing therapy in terms of OS but the 1-year survival rate did not show significant difference between two groups [35]. Additionally, statistically similar overall RR was observed in two meta-analyses [51, 52]. Another one found similar objective RR between two groups [35]. One meta-analysis did not report relevant data. Two meta-analyses reported PFS [35, 51]. One favored CPT-11-containing regimen [35] while the other showed no significant difference between two groups [51]. Besides, one meta-analysis reported TTF, which indicated that CPT-11-based therapy was favorable regarding TTF [52]. Treatment related death was reported in two meta-analyses. Both of them showed no significant difference between two regimens [51, 59]. Grade 3/4 fatigue was higher in CPT-11-based therapy according to the only related meta-analysis [35]. What's more, another meta-analysis revealed lower grade 3/4 hematological toxicity and gastrointestinal toxicity in CPT-11-containing therapy [52].

Only RCT studies, rather than non-RCTs were included in the four meta-analyses.

The meta-analysis by Qi had the largest number of included studies (*n* = 10) (Supplementary Table S16). Notably, there was an overlap of included studies among the four articles.

Given its superiority in the quantity of RC studies, the results of the meta-analysis by Qi might be more reliable. In details, it provided strong evidence for a survival benefit of CPT-11-containing regimen as first-line treatment for AGC patients. The survival benefit of CPT-11-based therapy for AGC was also confirmed in the other meta-analyses.

#### Platinum-based therapy *vs*. non platinum-based therapy

Platinum-based chemotherapy was evaluated in one meta-analysis. Non-platinum group were divided into two subgroups depending on whether new-generation agents such as S-1, taxol or CPT-11 were contained. Platinum-based therapy achieved better outcomes in terms of OS and RR compared with old-generation therapies. On the contrary, when comparing to non-platinum regimens containing new-generation agents, platinum-based therapy did not seem to achieve better survival and enhanced response. Besides, Platinum-based therapy was associated with higher risk of most hematological and non-hematological toxicity events. In addition, toxic death rate was statistically similar between Platinum-based and non Platinum-based regimens [28].

#### Cisplatin-based therapy *vs*. non Cisplatin-based therapy

Only one meta-analysis compared the outcomes of cisplatin-based therapy *versus* non cisplatin-based therapy. The results demonstrated that cisplatin-free combination therapy significantly enhanced OS, RR and PFS compared with cisplatin-based combination chemotherapy [33].

#### Targeted therapy

The efficacy of targeted therapy was evaluated in three meta-analyses [10, 18, 22]. As for OS, one of them favored targeted therapy of antiangiogenic and HER2 pathway but not for EGFR pathway compared with conventional chemotherapy [10]. One showed that in patients with Eastern Cooperative Oncology Group Performance Status (ECOG-PS) of 0, targeted therapy did not report a significant benefit over BSC. Contrarily, chemotherapy was associated with better survival compared to targeted therapy. In patients with ECOG-PS = 1 or 2, targeted therapy was superior to BSC in terms of OS. However, it did not show significant difference when comparing to chemotherapy [18]. Another meta-analysis that compared anti-VEGF therapy with non-anti-VEGF therapy favored anti-VEGF therapy regarding OS [22]. When it comes to RR, one of them favored anti-HER2 agents but not for anti-EGFR and anti-angiogenic agents [10]. One comparing anti-VEGF therapy with non-anti-VEGF therapy favored anti-VEGF therapy in terms of objective RR [22]. One meta-analysis did not reported relevant data. Only one meta-analysis evaluated PFS, which indicated that the targeted therapy of anti-angiogenic and HER2 pathway but not EGFR pathway was superior to conventional chemotherapy [10]. Also, only one meta-analysis reported recurrence-free survival (RFS) that anti-VEGF agents reported a significant benefit over non-anti-VEFG agents [22]. As for toxicity, one meta-analysis showed that the incidence of diarrhea and rash was higher in targeted therapy [10]; another found that grade 3 or 4 thrombocytopenia, diarrhea, and hypertension was significantly higher in anti-VEGF therapy compared to non-anti-VEFG therapy [22].

Only RCT studies, rather than non-RCTs were included in the three included meta-analyses.

The meta-analysis by Ciliberto had the largest number of included studies (*n* = 22) followed by the meta-analyses by Qi (*n* = 7) and by Iacovelli (*n* = 2) (Supplementary Table S17). Notably, there was an overlap of included studies between the meta-analyses by Iacovelli and by Ciliberto. All studies about targeted therapy that were included in the meta-analysis by Iacovelli were also covered in the meta-analysis by Ciliberto.

Given its superiority in the quantity of RCT studies, the results of the meta-analysis by Ciliberto might be more reliable. In details, targeted therapy showed a significant survival benefit, which could be ascribed to anti-angiogenic and anti-HER2 agents. Moreover, diarrhea occurrence was higher in anti-HER2 agents while rash occurrence was higher in anti-EGFR drugs.

#### Combination (doublet/triplet) therapy *vs*. single/doublet therapy

There were four meta-analyses to compare the outcomes of combination therapy, which included doublet and triplet therapy *versus* monotherapy or doublet therapy 8, 39, 51, 59]. Three of them came to the conclusion that the combination therapy was superior to single or doublet therapy in terms of OS [8, 39, 51] while the other one did not reported survival data. Notably, subgroup analyses based on treatment regimens in one of the meta-analyses showed that targeted agent plus cytotoxic chemotherapy significantly improved OS, but not for doublet cytotoxic chemotherapy [8]. As for RR, two of them showed that the combination therapy was favorable over single or doublet therapy [8, 51]. Similarly, subgroup analyses based on treatment regimens in one of the meta-analyses showed that targeted agent plus cytotoxic chemotherapy significantly improved objective RR, but not for doublet cytotoxic chemotherapy [8]. Another meta-analysis favored triplet combination therapy over doublet combination therapy in terms of overall RR [39]. One meta-analysis did not reported RR. As for PFS, the only one meta-analysis indicated that doublet combination therapy was associated with better PFS [8]. Besides, only one meta-analysis reported TTP which demonstrated that TTP was significantly improved by the use of combination therapy [51]. As for toxicity, three of them revealed that the toxicity of combination therapy was significantly higher compared to momotherapy or doublet therapy [8, 51, 59] while the other did not report toxicity in general. However, the incidence of grade 3 or 4 AEs was statistically similar between two groups according to one of the meta-analyses [39].

Only RCT studies, rather than non-RCTs were included in the four meta-analyses.

The meta-analysis by Wagner 2010 had the largest number of included studies (*n* = 13) followed by the meta-analyses by Liu (*n* = 12), Wagner 2006 (*n* = 11) and Zhang (*n* = 10) (Supplementary Table S18). Notably, there was an overlap of included studies among these four articles.

Given its superiority in the quantity of RCT studies, the results of the meta-analysis by Wagner 2010 might be more reliable. In details, combination chemotherapy improved survival compared to single-agent therapy. However, toxicity was higher in combination chemotherapy. In addition, the meta-analysis of Zhang indicated that no significant survival benefits were observed in doublet cytotoxic chemotherapy regimens while the addition of targeted agent to mono-chemotherapy as salvage treatment for pretreated AGC patients had substantial survival benefits.

#### FU/anthracycline-containing regimens with *vs*. without cisplatin

Two meta-analyses compared the outcomes of FU/anthracycline-containing therapy with *versus* without cisplatin. Both of them favored three-drug combination therapy. Other outcomes were not evaluated between the two groups [51, 59].

Only RCTs studies, rather than non-RCTs were included in the two meta-analyses.

The two meta-analyses shared completely the same included studies (*n* = 7) (Supplementary Table S19). Thus the results were completely consistent with each other. In details, they all agreed that best survival results were achieved with three-drug regimens containing FU, anthracycline, and cisplatin.

#### FU/cisplatin-containing regimens with *vs*. without anthracyclines

Two meta-analyses evaluated the outcomes of FU/cisplatin-containing regimens with *vs*. without anthracyclines. Both of them favor three-drug combination therapy. Other outcomes were not evaluated between the two groups [51, 59].

Only RCT studies, rather than non-RCTs were included in the two meta-analyses.

The number of studies included in these two articles was equal (*n* = 3) (Supplementary Table S20). The included studies were the same between the two meta-analyses and the results were completely consistent with each other. In details, they all agreed that best survival results were achieved with three-drug regimens containing FU, anthracycline, and cisplatin.

#### Docetaxel-containing *vs*. non-docetaxel-containing therapy

One meta-analysis focused on the efficacy of docetaxel-containing regimens. According to the study, Docetaxel containing therapy seemed not to be associated with significant better OS, objective RR and TTP compared to non-docetaxel based therapy. Besides, there were also no significant differences in treatment related death or treatment discontinuation due to toxicity between two groups [51].

#### Chemotherapy regimens with *vs*. without lentinan administration

Lentinan containing chemotherapy was evaluated in one meta-analysis. OS was enhanced by the use of the lentinan combination therapy [56]. Other outcomes were not reported in the study.

#### EPIPC *vs*. early postoperative intravenous chemotherapy

One meta-analysis compared EPIPC with early postoperative intravenous chemotherapy. EPIPC was associated with better 1-, 2-, 3-, and 5-year survival rate compared to early postoperative intravenous chemotherapy. Besides, 2- and 3-year intra-abdominal recurrence was significantly reduced by the use of early postoperative intravenous chemotherapy. Significant lower incidence of nausea and vomiting were observed in EPIPC group. Additionally, EPIPC was superior to intravenous chemotherapy in terms of liver and renal protection [54].

### Traditional chinese medicine (TCM)

#### Shenqifuzheng (SQFZ) injection plus chemotherapy *vs*. chemotherapy alone

Two meta-analyses compared the outcomes of SQFZ injection combined with chemotherapy *versus* chemotherapy alone [11, 25]. Only one meta-analysis reported overall RR, which showed that chemotherapy combined with SQFZ injection was favorable over chemotherapy alone [25]. Besides, one of them indicated that significant benefit was observed in SQFZ injection plus chemotherapy in terms of quality of life, complete and partial remission, and AEs compared with chemotherapy alone [11]. The other meta-analysis suggested that the Karnofsky score (KPS) was significantly higher in chemotherapy combined with SQFZ injection [25].

Only RCT studies, rather than non-RCTs were included in the meta-analysis by Li while the information regarding RCT studies was not available in the meta-analysis by Yao.

The meta-analysis by Yao had the largest number of included studies (*n* = 15) followed by the meta-analysis by Li (*n* = 13) (Supplementary Table S21). Notably, there was an overlap of included studies between them.

Given its superiority in the quantity of RCT studies, the results of the meta-analysis by Li might be more reliable. In details, SQFZ injection combined with chemotherapy could improve the clinical efficacy and performance status in patients with AGC.

#### Huachansu plus chemotherapy *vs*. chemotherapy alone

Only one meta-analysis evaluated the efficacy of Huachasu plus chemotherapy *versus* chemotherapy alone. There was no significant difference in 1-year OS between two groups. Besides, Huachasu plus chemotherapy was favorable over chemotherapy alone in term of total RR, KPS, gastrointestinal side effects, and leucocytopenia [37].

#### Compound matrine injection plus cisplatin therapy *vs*. cisplatin therapy alone

One meta-anaysis compared the outcomes between compound matrine injection plus cisplatin regimen *versus* cisplatin regimen alone. Quality of life and clinical efficacy were favorable for compound matrine injection combined with cisplatin therapy over cisplatin therapy alone. Additionally, compound matrine injection plus cisplatin therapy was associated with significantly lower incidence of leukopenia, thrombocytopenia, and gaastrointestinal AEs compared to cisplatin therapy alone [43].

#### Kanglaite (KLT) plus chemotherapy *vs*. chemotherapy alone

One meta-analysis evaluated the efficacy of KLT combined with chemotherapy regimen. KLT significantly improved 1-year OS compared to chemotherapy alone. KLT plus chemotherapy was associated with better quality of life and clinical efficacy compared with chemotherapy alone according to the results. Besides, KLT combined with chemotherapy regimen was superior to chemotherapy alone in terms of liver protection, incidence of cachexia and AEs [47].

## DISCUSSIONS

The treatment for AGC include surgery gasterectomy, perioperative chemotherapy with or without chemoradiation, palliative chemotherapy, targeted therapy BSC and other.

Surgery remains the most important component of curative therapy [61]. According to the JGCA treatment guidelines, distal gastrectomy with D2 lymphadenectomy via open approach is the standard procedure for AGC [5]. It was suggested in our study that LG was a safe and technical alternative to OG for AGC patients with a lower complication rate and enhanced postoperative recovery. The results were consistent with recent multicenter RCT and research [62,63]. Besides, NAC and AC were thought to benefit the survival over surgery alone though no clear superiority of one strategy over another has emerged [41, 57]. In metastatic setting, chemotherapy is the mainstay treatment. And it was demonstrated in the present study that targeted therapy like anti-angiogenic and anti-HER2 agents but anti-EGFR agents might have a significant survival benefit [10]. Notably, it was suggested that addition of TCM such as SQFZ injection to chemotherapy could improve clinical efficacy and benefit the quality of life compared to chemotherapy alone [11, 37].

### Limitations

We recognize that the present review has some limitations. First, not all the related survival and clinical data were evaluated in every meta-analysis. As a result, made it difficult to compare directly between meta-analyses. Second, the accuracy of the findings in every meta-analysis could not be guaranteed since it was not possible for us to repeat every meta-analysis and the heterogeneity among included studies in every meta-analysis were not considered. Third, there was overlap of the original studies in each meta-analysis; and we arbitrarily evaluated the reliability of meta-analysis according to the quality and quantity of the included meta-analysis. Fourth, conversion therapy, immune therapy and radiation therapy were not discussed in the present study.

### Recommendations

#### Surgery

1. LG is a safe and technical alternative to OG for AGC patients with a lower complication rate and enhanced postoperative recovery (*grade of recommendation: moderate*).
2. NAC can improve the tumor resection rate and the survival rate in AGC patients without increasing the operative risk and perioperative mortality (*grade of recommendation: high*).
3. Postoperative chemotherapy can improve OS after radical surgery for gastric cancer (*grade of recommendation: high*).
4. IPC has positive effect on overall and peritoneal recurrence and distant metastasis. Besides, loco-regional lymph-nodes invasion in patients affected by AGC is not a contraindication to IPC (*grade of recommendation: high*).
5. Palliative gastrectomy for patients with incurable AGC was associated with longer OS, especially for patients with stage M1 gastric cancer (*grade of recommendation: low*).
6. Palliative gastrectomy combined with hepatectomy might provide better OS than palliative gastrectomy only (*grade of recommendation: low*).
7. Avoiding the use of abdominal drains might reduce drain-related complications and shortened hospital stay after gastrectomy (*grade of recommendation: high*).

#### Chemotherapy

1. Chemotherapy significantly improved survival in comparison to BSC (*grade of recommendation: high*).
2. S-1-based regimens are effective and tolerable as first-line treatment of AGC in both Asian and Western countries though the toxicity profile of S-1 was clearly more advantageous in Western patients (*grade of recommendation: high*).
3. S-1 and capecitabine could be used interchangeably for AGC, at least in Asia (*grade of recommendation: high*).
4. S-1 combination therapy was more efficacious than S-1 monotherapy though AEs was higher and safety profile was poorer in S-1 combination therapy (*grade of recommendation: high*).
5. Oxaliplatin-based therapy was associated with a small but significant survival benefit with less toxicity and better tolerability (*grade of recommendation: moderate*).
6. CPT-11-containing regimen had survival benefit as first-line treatment for AGC patients (*grade of recommendation: high*).
7. New-generation agent such as S-1, taxanes and irinotecan seemed to be valid options for patients with inoperable AGC as first-line chemotherapy (*grade of recommendation: high*).
8. Replacing cisplatin with oxaliplatin, CPT-11, or taxane significantly enhanced OS, RR and PFS compared with cisplatin-based combination chemotherapy (*grade of recommendation: high*).
9. Targeted therapy may have a significant survival benefit, which could be ascribed to anti-angiogenic and anti-HER2 agents (*grade of recommendation: high*).
10. Combination chemotherapy may improve survival compared to single-agent therapy at a price of higher toxicity (*grade of recommendation: high*).
11. Better survival results should be achieved in FU, anthracycline and cisplatin combination therapy no matter comparing to FU/anthracycline doublet regimens FU/or FU/cisplatin doublet regimens (g*rade of recommendation: high*).
12. The addition of lentinan to standard chemotherapy may offer a significant advantage over chemotherapy alone in terms of survival (g*rade of recommendation: high*).
13. EPIPC may improve survival rate and reduce both recurrence rate and side effects (g*rade of recommendation: high*).

#### TCM

1. SQFZ injection combined with chemotherapy could improve the clinical efficacy and performance status in patients with AGC (g*rade of recommendation: high*).
2. Huchansu combined with chemotherapy provides benefits for AGC on improving the response rate, increasing Karnofsky score, reducing leucocytopenia and major side effects (g*rade of recommendation: high*).
3. Compound matrine injection combined with cisplatin chemotherapy can improve the quality of life with lower AEs (g*rade of recommendation: high*).

#### Uncertainties

1. D4 lymphadenectomy should be performed prudently for its wound degree of surgery is significantly higher. Standard operating procedures should be established to reduce wound degree.
2. The evidence is limited to confirm that XELOX may share similar efficacy as FOLFOXs and reduce toxicities.
3. Though the addition of docetaxel to the cisplatin/fluorouracil combination may provide survival benefit, the clinical value of this regimen is regarded as controversial for its significant toxicity.
4. The benefit of KLT plus chemotherapy may be confirmed in further rigorously controlled trials.

## MATERIALS AND METHODS

### Search strategy and study selection

All meta-analysis papers regarding the treatment of AGC via the PubMed, Google Scholar, and Web of Science were retrieved. The search strategy terms used in the English databases were “treatment OR management OR therapy” AND “advanced gastric cancer OR advanced gastric carcinoma OR advanced gastric neoplasm OR advanced stomach cancer OR advanced stomach carcinoma OR advanced stomach neoplasm” AND “meta-analyses”. The last search was performed on March, 13, 2016.

Eligibility criteria were as follows. 1) All meta-analyses regarding the treatment of AGC. 2) Duplicate publications. 3) Only abstract available. 4) Meeting abstracts. 5) Only systematic reviews without meta-analyses. 6) Patients without AGC. 7) Other topics, but not treatment modalities.

Primary outcomes were OS, RR, DFS, RFS, PFS TTP, TTF and other endpoints.

### Reliability of meta-analyses

As the results were different among the meta-analyses, the reliability was evaluated according to the quality and quantity of original studies included in every meta-analysis. First, the results of a meta-analysis would be more reliable if a larger number of RCTs were included. And if the larger number of patients were included in the RCTs, the results of a meta-analysis would be more reliable. Second, the number of non-RCT studies was further evaluated if the number of RCTs and patients were similar. Third, if the number of RCTs and non-RCT studies included was similar but the results were not consistent among meta-analyses, the total number of the included patients and statistical methods would be further evaluated. If the hazard ratio was calculated, the results of a meta-analysis would be more reliable.

### Grade of recommendations

Grade of recommendation was determined in the way that was suggested by a previous study [64]. High grade recommendation was considered if the results of meta-analyses were based on more than 3 single-center RCTs or 1 multi-center RCT. Low grade recommendation was considered, if the results of meta-analyses were based on the non-RCT studies alone. As for something in between, moderate grade recommendation was considered.

## SUPPLEMENTARY MATERIALS TABLES



## Abbreviations

AGC: advanced gastric cancer
LG: laparoscopic gastrectomy
OG: open gastrectomy
BSC: basic supportive care
RCT: randomized controlled trial
NAC: neoadjuvant chemotherapy
AC: adjuvant chemotherapy
IPC: intraperitoneal chemotherapy
HIIPC: hyperthermic intraoperative intraperitoneal chemotherapy
EPIPC: early postoperative intraperitoneal chemotherapy
NIIPC: normothermic intraoperative intraperitoneal chemotherapy
DPIPC: delayed postoperative intraperitoneal chemotherapy
CPT-11: irinotecan
TCM: Traditional Chinese Medicine
SQFZ: Shenqifuzheng
KLT: Kanglaite
OS: overall survival
RR: response rate
DFS: disease-free survival
RFS: recurrence-free survival
PFS: progression-free survival
TTP: time-to-progression
TTF: time-to failure.
