# Supplementary material for: Management of advanced gastric cancer: An overview of major findings from meta-analysis
Source: Oncotarget. 2016 Sep 17;7(47):78180–205. doi: 10.18632/oncotarget.12102 (PMC5363654; doi:10.18632/oncotarget.12102)
Supplement: Supplementary file 1 [file oncotarget-07-78180-s001.docx]

**Supplementary Table 1. Study characteristics: An overview of included studies**

| **First Author** | **Journal (Year)** | **Country** | **Type of participants** | **No. included studies** | **No. included patients** | **Comparisons** | **Type of Studies** | |
| --- | --- | --- | --- | --- | --- | --- | --- | --- |
|  |  |  |  |  |  |  | **RCT** | **Non-RCT** |
| Ter Veer E | Gastric Cancer (2016) | Netherlands | AGC patients | 18 | 4883 | S-1-based therapy vs. 5-FU; S-1-based therapy vs. capecitabine-based therapy; S-1-based combination therapy vs. S-1 monotherapy | 18 | 0 |
| Zhang Y | Medicine (Baltimore) (2016) | China | AGC patients | 10 | 1698 | Doublet combination therapy vs. single therapy as second-line treatment | 10 | 0 |
| Badiani B | World J Clin Oncol (2015) | Italy | AGC patients | 7 | 2298 | Six chemotherapy vs.BSC | 7 | 0 |
| Ciliberto D | Cancer Biol Ther (2015) | Italy | AGC patients | 22 | 7022 | Targeted therapy vs. conventional therapy | 22 | 0 |
| Li J | Chin J Integr Med (2015) | China | AGC patients | 13 | 860 | SQFZ injection+ chemotherapy vs. chemotherapy alone | 13 | 0 |
| Lu C | Surg Endosc (2015) | China | AGC patients | 8 | 1364 | LADG vs. ODG | 0 | 8 |
| Quan Y | Gastric Cancer (2015) | China | AGC patients | 26 | NA | LG vs. OG | NA | NA |
| Wu FL | Medicine (Baltimore) (2015) | China | AGC patients | 15 | 2973 | S-1-based therapy vs. 5-FU therapy; S-1-based therapy vs. capecitabine- based therapy | 12 | 3 |
| Xu HB | Eur J Clin Pharmacol (2015) | China | AGC patients | 26 | 1585 | Capecitabine+ oxaliplatin vs. 5-fluorouracil/leucovorin+ oxaliplatin | 26 | 0 |
| Coccolini F | Eur J Surg Oncol (2014) | France | AGC patients | 20 | 2145 | IPC + surgery vs. surgery alone | 20 | 0 |
| Huang YL | Int J Clin Exp Med (2014) | China | AGC patients | 11 | 1904 | LAG vs. OG | NA | NA |
| Iacovelli R | PLoS One (2014) | Italy | AGC patients | 5 | 1407 | Targeted therapy vs. BSC or chemetherapy | NA | NA |
| Li DH | Tumour Biol (2014) | China | AGC patients | 6 | 2264 | S-1-based vs. 5-FU-based chemotherapy | 6 | 0 |
| Liu GF | World J Gastroenterol (2014) | China | AGC patients | 4 | 790 | S-1-based combination therapy vs. S-1 monotherapy | 4 | 0 |
| Liu H | Medicine (Baltimore) (2014) | China | AGC patients | 3 | 352 | Paclitaxel + S-1 vs. paclitaxel + 5-FU | 3 | 0 |
| Qi WX | Tumour Biol (2014) | China | AGC patients | 7 | 2340 | Anti-VEGF agents vs. non anti-VEGF agents | 7 | 0 |
| Wu JR | Tumour Biol (2014) | China | AGC patients | 6 | 913 | S-1-based therapy vs. S-1 monotherapy | 5 | 1 |
| Yang J | World J Gastroenterol (2014) | China | AGC patients | 7 | 2176 | S-1-based vs. non-S-1-based chemotherapy | 7 | 0 |
| Yao K | J Cancer Res Ther (2014) | China | AGC patients | 15 | 1621 | SQFZ injection + chemotherapy vs. chemotherapy alone | NA | NA |
| Zou ZH | World J Gastroenterol (2014) | China | AGC patients | 14 | 2596 | LGD2 vs. OGD2 | 1 | 13 |
| Chen K | World J Surg Oncol (2013) | China | AGC patients | 15 | NA | LG vs. OG | 2 | 13 |
| Chen WW | PLoS One (2013) | China | Inoperable AGC patients | 27 | 3680 | Platinum vs. non-platinum chemotherapy as first-line palliative treatment | 27 | 0 |
| Choi YY | J Surg Oncol (2013) | Korea | AGC patients | 10 | 1819 | LG vs. OG | 1 | 9 |
| He MM | PLoS One (2013) | China | AGC patients | 10 | 821 | S-1-based chemotherapy vs. capecitabine-based chemotherapy as first-line treatment | 5 | 5 |
| Kim HS | Ann Oncol (2013) | Korea | AGC patients | 3 | 410 | Second-line chemotherapy vs. BSC | 3 | 0 |
| Mi DH | Int J Hyperthermia (2013) | China | Resectable locally AGC | 16 | 1906 | Surgery with vs. without IHIPC | 16 | 0 |
| Petrelli F | PLoS One (2013) | Italy | AGC patients | 14 | 2981 | Chemotherapy with vs. without cisplatin | 14 | 0 |
| Qiu J | Surg Laparosc Endosc Percutan Tech (2013) | China | AGC patients | 7 | 1271 | LADG vs. ODG | 0 | 7 |
| Qi WX | Int J Cancer (2013) | China | AGC patients | 10 | 1837 | Irinotecan-containing regimens vs. nonirinotecan-contining regimen as first-line treatment | 10 | 0 |
| Sun J | BMC Cancer (2013) | China | Incurable AGC patients | 14 | 3003 | Palliative gastrectomy vs. non-palliative gastrectomy | 0 | 14 |
| Xie X | Med Hypotheses (2013) | China | AGC patients | 15 | 1008 | Huachansu combined with chemotherapy vs. chemotherapy alone | 15 | 0 |
| Ye LY | J Zhejiang Univ Sci B (2013) | China | AGC patients | 7 | 815 | LAG vs. OG | 1 | 6 |
| Liu N | Chinese Journal of Hospital Pharmacy (2012) | China | AGC patients | 12 | NA | Triplet combination chemotherapy vs. doublet combination chemotherapy | 12 | 0 |
| Ma Y | J Clin Pharm Ther (2012) | China | AGC patients | 18 | 2175 | Capecitabine-based chemotherapy vs.5-FU-based treatments | 18 | 0 |
| Xiao F | Chongqing Medicine (2012) | China | AGC patients | 18 | 2685 | NAC vs. surgery alone | 18 | 0 |
| Huang J | Med Oncol (2011) | China | AGC patients | 4 | 2115 | S-1-based therapy vs. fluorouracil (5-FU)-based therapy | 4 | 0 |
| Huang S | Zhongguo Zhong Yao Za Zhi (2011) | China | AGC patients | 10 | 710 | Compound matrine injection + chemotherapy vs. cisplatin chemotherapy | 10 | 0 |
| Liu HP | Dig Surg (2011) | China | AGC patients | 4 | 438 | Drain vs. no-drain after gastrectomy | 4 | 0 |
| Martinez-Ramos D | Rev Esp Enferm Dig (2011) | Spain | AGC patients | 7 | 2344 | LG vs. OG | 1 | 6 |
| Montagnani F | Gastric Cancer (2011) | Italy | Unresectable AGC patients | 3 | 1294 | Oxaliplatin vs. cisplatin | 3 | 0 |
| Wang C | Modern Journal of Integrated Traditional Chinese and Western Medicine (2011) | China | AGC patients | 4 | 304 | KLT+chemotherapy vs. chemotherapy alone | 4 | 0 |
| Zhang L | Chinese General Practice (2011) | China | AGC patients | 5 | 1685 | D2 vs. D3 dissection | 1 | 4 |
| Zhang YL | Chinese Journal of General Surgery. (2011) | China | AGC patients | 9 | 1171 | EPIPC vs. early postoperative intravenous chemotherapy | 9 | 0 |
| Li W | World J Gastroenterol (2010) | China | AGC patients | 14 | 2271 | NAC vs. no therapy before surgery | NA | NA |
| Wagner AD | Cochrane Database Syst Rev (2010) | Switzerland | AGC patients | 35 | 5726 | Chemotherapy vs.BSC; combination vs. single-agent chemotherapy; 5-FU/cisplatin-containing combination therapy regimens with vs. without anthracyclines; 5-FU/anthracycline-containing combinations with vs. without cisplatin; Irinotecan vs. non-irinotecan; docetaxel vs. non-docetaxel containing regimens. | 35 | 0 |
| Wang DL | World J Gastroenterol (2010) | China | AGC patients | 4 | 657 | CPT-11-containing combination chemotherapy vs. non CPT-11-containing combination chemotherapy | 4 | 0 |
| Wang Z | World J Gastroenterol (2010) | China | AGC patients | 8 | 2021 | D2 + para-aortic nodal dissection (D4) vs. D2 lymphadenectomy | 4 | 4 |
| Zhang YL | Chin J Gen Surg(2010) | China | AGC patients | 4 | 1120 | D2 vs. D4 dissection | 4 | 0 |
| Gong JF | Zhonghua Yi Xue Za Zhi (2009) | China | AGC patients | 16 | 2121 | Oxaliplatin-based chemotherapy vs. cisplatin-based chemotherapy | NA | NA |
| Oba K | Anticancer Res (2009) | Japan | Unresectable/recurrent AGC patients | 5 | 650 | Chemotherapy regimens with vs. without lentinan | 5 | 0 |
| Sun P | Br J Surg (2009) | China | AGC patients | 12 | 3809 | Surgery with chemotherapy vs. surgery only | 12 | 0 |
| Yan TD | Ann Surg Oncol (2007) | Australia | Locally resectable AGC patients | 13 | 1648 | Surgery with vs. without IPC | 13 | 0 |
| Wagner AD | J Clin Oncol (2006) | Germany | AGC patients | 20 | 4198 | Chemotherapy vs. BSC; combination vs. single agent; FU/cisplatin-containing regimens with vs. without anthracyclines; FU/anthracycline-containing combinations with vs. without cisplatin; irinotecan-containing vs. nonirinotecan-containing combinations (mainly FU/cisplatin) | 20 | 0 |
| Casaretto L | Braz J Med Biol Res (2006) | Brazil | Unresectable AGC patients | 5 | 390 | Chemotherapy vs. BSC | 5 | 0 |

**Supplementary Table S2. Findings of meta-analyses: Other endpoints (supplement to Table 1)**

| **First Author** | **Journal (Year)** | **Comparisons** | **Other** |
| --- | --- | --- | --- |
|  |  |  |  |
| **LG vs. OG** | | | |
| Lu C[12] | Surg Endosc(2015) | LADG vs. ODG | Number of harvested lymph nodes: statistically similar. |
| Quan Y[13] | Gastric Cancer (2015) | LG vs. OG | Blood loss: less in LG. Hospitalization: shorter in LG. Bowel recovery, ambulation: faster in LG.  Complications: fewer in LG. Number of harvested lymph nodes: statistically similar. |
| Huang YL[17] | Int J Clin Exp Med(2014) | LAG vs. OG | Blood loss: less in LAG. Time to flatus, hospital stay: shorter in LAG. Operating time: longer in LAG. Complications: less in LAG. Number of harvested lymph nodes, hospital mortality: statistically similar. |
| Zou ZH[26] | World J Gastroenterol (2014) | LGD2 vs. OGD2 | Blood loss, analgesic consumption, postoperative morbidity: lower in LGD2. Times to first ambulation, flatus, and oral intake, hospitalization: shorter in LGD2. Operative times: longer in LGD2. Reoperation incidence, postoperative mortality number of harvested lymph nodes: statistically similar. |
| Chen K[27] | World J Surg Oncol (2013) | LG vs. OG | Operating time: longer in LG. Blood loss: less in LG. Time to flatus, hospital stay: shorter in LG. Complications: fewer in LG. Number of harvested lymph nodes: statistically similar. |
| Ye LY[38] | J Zhejiang Univ Sci B (2013) | LAG vs. OG | Blood loss, analgesics comsumption: less in LAG. Time to flatus, hospital stay: shorter in LAG. Operating time: longer in LAG. Complications, harvested lymph nodes, overall mortality, cancer-related mortality: statistically similar. |
| Qiu J[34] | Surg Laparosc Endosc Percutan Tech (2013) | LADG vs. ODG | Operating time: longer in LADG. Blood loss, analgesic requirement: less in LADG. Hospital stay: shorter in LAG. Harvested lymph nodes, overall complications, postoperative mortality: statistically similar. |
| Choi YY[29] | J Surg Oncol (2013) | LG vs. OG | NA. |
| Martinez-Ramos D[45] | Rev Esp Enferm Dig (2011) | LG vs. OG | Operating time: longer in LG. Blood loss: less in LG. Hospital stay: shorter in LG. Harvested lymph nodes, cancer-related mortality risk: statistically similar. |

**Supplementary Table S3. Findings of meta-analyses: Other endpoints (supplement to Table 6)**

| **First Author** | **Journal (Year)** | **Comparisons** | **Other** |
| --- | --- | --- | --- |
| **Chemotherapy vs. BSC** | | |  |
| Badiani B[9] | World J Clin Oncol (2015) | 6 chemotherapy vs. BSC | NA. |
| Iacovelli R[18] | PLoS One (2014) | (Include chemotherapy vs. BSC) | NA. |
| Kim HS[31] | Ann Oncol (2013) | Second-line chemotherapy (irinotecan or docetaxel) vs. BSC | NA. |
| Wagner AD[51] | Cochrane Database Syst Rev (2010) | (Include chemotherapy vs. BSC). | NA. |
| Casaretto L[60] | Braz J Med Biol Res (2006) | Chemotherapy vs. BSC | Symptom-free period, quality of life, tumor mass reduction: favor chemotherapy. |
| Wagner AD[59] | J Clin Oncol (2006) | (include chemotherapy vs. BSC). | NA. |

**Supplementary Table S4. Findings of meta-analyses: Other endpoints (supplement to Table 7)**

| **First Author** | **Journal (Year)** | **Comparisons** | **Other** |
| --- | --- | --- | --- |
| **S-1-based therapy vs. 5-FU-based therapy** | | |  |
| Ter Veer E[7] | Gastric Cancer (2016) | S-1-based therapy vs. non S-1-based therapy (include S-1-based therapy vs. 5-FU based therapy) | Febrile neutropenia, toxicity-related deaths, grade 3–4 stomatitis and mucositis, grade 1–2 diarrhea, stomatitis, alopecia (in Western patients): lower in S-1-based therapy. Grade 3–4 fatigue and grade 1–2 abdominal pain (Asian): higher in S-1-based therapy. Grade 1–2 neutropenia, nausea and weight loss (Asian): lower in S-1-based therapy. Febrile neutropenia, serious AEs, toxicity-related deaths (Asian): statistically similar. |
| Wu FL[14] | Medicine (Baltimore) (2015) | S-1-based therapy vs. non S-1-based therapy (include S-1-based therapy vs. 5-FU therapy). | Overall grade 3-4 toxicity: statistically similar. Grade 3-4 toxicity of neutropenia, nausea: lower in S-1-based therapy. Grade 3-4 toxicity of diarrhea: higher in S-1-based therapy. |
| Yang J[24] | World J Gastroenterol (2014) | S-1-based therapy vs. non S-1-based therapy (include S-1-based therapy vs. 5-FU therapy). | Leukopenia and stomatitis: lower in S-1-based therapy. |
| Li DH[19] | Tumour Biol (2014) | S-1-based versus 5-FU based chemotherapy | Grade 3-4 toxicities of thrombocytopenia and stomatitis: lower in S-1-based therapy. |
| Liu H[21] | Medicine (Baltimore) (2014) | S-1+ paclitaxel vs. 5-FU+ paclitaxel | SD (stable disease): statistically similar. PD (progressive disease): lower in S-1+ paclitaxel therapy. Disease control rate: favor S-1+ paclitaxel therapy; Nausea, vomiting: lower in S-1+ paclitaxel therapy. |
| Huang J[42] | Med Oncol (2011) | S-1-based therapy vs. 5-FU-based therapy | Grade 3/4 neutropenia: lower in S-1 therapy. Grade 3/4 anemia, leucopenia, stomatitis, diarrhea, nausea, treatment-related death: statistically similar. |

**Supplementary Table S5: Overlap of included studies among meta-analyses regarding LG versus open gastrectomy**

| **First author** | **Martinez-Ramos D** | **Choi YY** | **Qiu J** | **Chen K** |
| --- | --- | --- | --- | --- |
| Journal (Year) | REV ESP ENFERM DIG (Madrid) (2011) | Journal of Surgical Oncology (2013) | Surg Laparosc Endosc Percutan Tech (2013) | World Journal of Surgical Oncology (2013) |
| Publication type | Full text | Full text | Full text | Full text |
| No. Included studies | 7 | 10 | 7 | 15 |
| No. Included RCTs | 1 | 1 | 0 | 2 |
| Included studies | Huscher CGS, et al. Ann Surg 2005;241:232-7. | Sica GS, et al. World J Gastroenterol 2011;17:4602–4606. | Hwang SI, et al. Surg Endosc. 2009;23:1252–1258. | Huscher CGS, et al. Ann Surg 2005;241:232-7. |
|  | Dulucq JL, et al. Surg Endosc 2005;19:933-8. | Shuang JB, et al. Gastrointest Surg 2011;15:57–62. | Shuang JB, et al. J Gastrointest Surg. 2011;15:57–62. | Shuang JB, et al. J Gastrointest Surg. 2011;15:57–62. |
|  | Weber KJ, et al. Surg Endos 2003;17:968-71. | Hamabe A, et al. Surg Endosc 2012;26:1702–1709. | XH DU, et al. Chin Med J. 2009;122:1404–1407. | XH DU, et al. Chin Med J. 2009;122:1404–1407 |
|  | Ziqiang W, et al. Surg Endosc 2006;20:1738-43. | Kim KH, et al. Dig Surg 2012;29:165–171. | Hoon H, et al. Surg Oncol. 2008;98:515–519. | Kim KH, et al. Dig Surg 2012;29:165–171. |
|  | Varela JE, et al. Am J Surg 2006;192:837-42. | Scatizzi M, et al. Updates Surg 2011;63:17–23. | Scatizzi M, et al. Updates Surg. 2011;63:17–23. | Scatizzi M, et al. Updates Surg. 2011;63:17–23. |
|  | Pugliese R, et al. Surg Endosc 2007;21:21-7. | Moisan F, et al. Surg Endosc 2012;26:661–672. | Huang JL, et al. Dig Surg. 2010;27:291–296. | Moisan F, et al. Surg Endosc 2012;26:661–672. |
|  | Strong VE, et al. Ann Surg Oncol 2009;16:1507-13. | Zhao Y, et al. Surg Endosc 2011;25:2960–2966. | Zhao YL, et al. Surg Endosc. 2011;25:2960–2966. | Zhao YL, Surg Endosc. 2011;25:2960–2966. |
|  |  | Maclellan SJ, et al. Surg Endosc 2012;26:1813–1821. |  | Du J, et al. Hepatogastroenterology 2010, 57:1589–1594. |
|  |  | Shinohara T, et al. Surg Endosc 2013;27:286–294. |  | Chen QY, et al. World J Surg Oncol 2012, 10:248. |
|  |  | Cai J, et al. Dig Surg 2011;28:331–337. |  | Cai J, et al. Dig Surg 2011;28:331–337. |
|  |  |  |  | Chun HT, et al. Yonsei Med J 2012, 53:952–959. |
|  |  |  |  | Siani LM, et al. Minerva Chir 2012, 67:319–326. |
|  |  |  |  | Shinohara T, et al. Surg Endosc 2013, 27:286–294. |
|  |  |  |  | Hwang SI, et al. Surg Endosc 2009, 23:1252–1258. |
|  |  |  |  | Hur H, et al. J Surg Oncol 2008, 98:515–519. |

**Overlap of included studies among meta-analyses regarding LG versus open gastrectomy (continued)**

| **First author** | **Ye LY** | **Zou ZH** | **Huang YL** | **Quan Y** | **Lu C** |
| --- | --- | --- | --- | --- | --- |
| Journal (Year) | J Zhejiang Univ-Sci B (Biomed & Biotechnol) (2013) | World J Gastroenterol (2014) | Int J Clin Exp Med (2014) | Gastric Cancer (2015) | Surg Endosc (2015) |
| Publication type | Full text | Full text | Full text | Full text | Full text |
| No. Included studies | 7 | 14 | 11 | 26 | 8 |
| No. Included RCTs | 1 | 1 | NA | 1 | 0 |
| Included studies | XH DU, et al. Chin Med J. 2009;122:1404–1407. | Hur H, et al. J Surg Oncol 2008, 98:515–519. | Hur H, et al. J Surg Oncol 2008, 98:515–519. | Lee SI, et al. J Am Coll Surg. 2006;202:874–80. | Huscher CGS, et al. Ann Surg 2005;241:232-7. |
|  | Shuang JB, et al. J Gastrointest Surg. 2011;15:57–62. | DU XH, et al. Chin Med J (Engl) 2009; 122:1404-1407 | DU XH, et al. Chin Med J (Engl) 2009; 122:1404-1407 | Hur H, et al. J Surg Oncol 2008, 98:515–519. | Ziqiang W, et al. Surg Endosc 2006;20:1738-43. |
|  | Huang JL, et al. Dig Surg. 2010;27:291–296. | Du J, et al. Hepatogastroenterology 2010, 57:1589–1594. | Huang JL, et al. Dig Surg. 2010;27:291–296. | Hwang SI, et al. Surg Endosc 2009, 23:1252–1258. | Hur H, et al. J Surg Oncol 2008, 98:515–519. |
|  | Cai J, et al. Dig Surg 2011;28:331–337. | Huang JL, et al. Dig Surg. 2010;27:291–296. | Cai J, et al. Dig Surg 2011;28:331–337. | Du J, et al. Hepatogastroenterology 2010, 57:1589–1594. | DU XH, et al. Chin Med J (Engl) 2009; 122:1404-1407 |
|  | Hamabe A, et al. Surg Endosc 2012;26:1702–1709. | Cai J, et al. Dig Surg 2011;28:331–337. | Scatizzi M, et al. Updates Surg. 2011;63:17–23. | Cai J, et al. Dig Surg 2011;28:331–337. | Huang JL, et al. Dig Surg. 2010;27:291–296. |
|  | Hwang SI, et al. Surg Endosc 2009, 23:1252–1258. | Scatizzi M, et al. Updates Surg. 2011;63:17–23. | Shuang JB, et al. J Gastrointest Surg. 2011;15:57–62. | Jeong SH, et al. Surg Endosc. 2011;25:872–8. | Scatizzi M, et al. Updates Surg. 2011;63:17–23. |
|  | Hur H, et al. J Surg Oncol 2008, 98:515–519. | Shuang JB, et al. J Gastrointest Surg. 2011;15:57–62. | Kim KH, et al. Dig Surg 2012;29:165–171. | Scatizzi M, et al. Updates Surg. 2011;63:17–23. | Shuang JB, et al. J Gastrointest Surg. 2011;15:57–62. |
|  |  | Zang WD, et al. Zhonghua Zhong Liu Zazhi 2011; 33: 864-867 | Hamabe A, et al. Surg Endosc 2012;26:1702–1709. | Shuang JB, et al. J Gastrointest Surg. 2011;15:57–62. | Zhao YL, Surg Endosc. 2011;25:2960–2966. |
|  |  | Chen QY, et al. World J Surg Oncol 2012, 10:248. | Chen QY, et al. World J Surg Oncol 2012, 10:248. | Sica GS, et al. World J Gastroenterol. 2011;17:4602–6. | Chun HT, et al. Yonsei Med J 2012, 53:952–959. |
|  |  | Hamabe A, et al. Surg Endosc 2012;26:1702–1709. | Shinohara T, et al. Surg Endosc 2013, 27:286–294. | Zhao YL, Surg Endosc. 2011;25:2960–2966. | Cui M, et al. World J Gastroenterol 2012:18:833–839 |
|  |  | Sato H, et al. Surg Endosc 2012; 26: 2240-2246 | Lin JX, et al. J Surg Oncol 2013; 11: 4. | Chen QY, et al. World J Surg Oncol 2012, 10:248. |  |
|  |  | Wang DR, et al. Zhonghua Wei Chang Wai Ke Zazhi 2012; 15: 964-966 |  | Chun HT, et al. Yonsei Med J 2012, 53:952–959. |  |
|  |  | Kim KH, et al. Dig Surg 2012;29:165–171. |  | Hamabe A, et al. Surg Endosc 2012;26:1702–1709. |  |
|  |  | Shinohara T, et al. Surg Endosc 2013, 27:286–294. |  | Kim KH, et al. Dig Surg 2012;29:165–171. |  |
|  |  |  |  | MacLellan SJ, et al. Surg Endosc. 2012;26:1813–21. |  |
|  |  |  |  | Moisan F, et al. Surg Endosc 2012;26:661–672. |  |
|  |  |  |  | Sato H, et al. Surg Endosc 2012; 26: 2240-2246 |  |
|  |  |  |  | Bo T, et al. J Gastrointest Surg 2013;17:1202–1208. |  |
|  |  |  |  | Fang F, et al. Exp Ther Med. 2013;6:753–8. |  |
|  |  |  |  | Gordon AC, et al. Surg Endosc. 2013;27:462–70. |  |
|  |  |  |  | Li ZX, et al. J BUON. 2013;18:689–94. |  |
|  |  |  |  | Lin JX, et al. World J Surg Oncol.2013;11:4. |  |
|  |  |  |  | Shinohara T, et al. Surg Endosc. 2013;27:286–94. |  |
|  |  |  |  | Yamanaka N, et al. Surg Today. 2013;43:859–64. |  |
|  |  |  |  | Fang C, Hua J, et al. Am J Surg. 2014;208:391–6. |  |
|  |  |  |  | Lee JH, et al. Surgery (St. Louis). 2014;155:154–64. |  |

**Supplementary Table S6: Overlap of included studies among meta-analyses regarding NAC vs. no therapy before surgery**

| **First author** | **Li W** | **Xiao F** |
| --- | --- | --- |
| Journal (Year) | World J Gastroenterol (2010） | Chongqing Medicine (2012) |
| Publication type | Full text | Full text |
| No. Included studies | 14 | 18 |
| No. Included RCTs | NA. | 18 |
| Included studies | Schuhmacher C, et al. J Clin Oncol (Meeting Abstracts) 2009; 27 (15S): 4510 | Biffi R, et al. World J Gastroenterol,2010,16(7):868-874 |
|  | Boige V, et al. Proc Am Soc Clin Oncol 2007; 25 (18S): 4510 | LinMB, et al. Fujian Medical Journal,2010,32(5)：20-22 |
|  | Cunningham D, et al. N Engl J Med 2006; 355: 11-20 | Zhou K, et al. Jiangsu Medical Journal,2010,26(19):2333-2334 |
|  | Hartgrink HH, et al. Eur J Surg Oncol 2004; 30: 643-649 | Song YQ, et al. Medical Journal of Qilu,2009,24(5):385-387 |
|  | Nio Y, et al. Anticancer Res 2004; 24: 1879-1887 | Schuhmacher C, et al. J Clin Oncol,2010,28(35):5210-5218 |
|  | Zhang CW, et al. World J Gastroenterol 2004; 10: 3070-3072 | Boige V, et al. Proc Am Soc Clin Oncol 2007; 25 (18S): 4510 |
|  | Kobayashi T, et al. Gan To Kagaku Ryoho 2000; 27: 1521-1526 | Cunningham D, et al. N Engl J Med 2006; 355: 11-20 |
|  | Wang XL, et al. Oncol Rep 2000; 7: 241-244 | Hartgrink HH, et al. Eur J Surg Oncol 2004; 30: 643-649 |
|  | Takiguchi N, et al. Proc Am Soc Clin Oncol 2000; 19: A1178 | Nio Y, et al. Anticancer Res 2004; 24: 1879-1887 |
|  | Lygidakis NJ, et al. Hepatogastroenterology 1999; 46: 2035-2038 | Zhang CW, et al. World J Gastroenterol 2004; 10: 3070-3072 |
|  | Kang YK, et al. Abstract 503 presented at the ASCO Annual Meeting, 1996. Available from: URL: <http://www>. asco.org/ASCOv2/Meetings/Abstracts?&vmview=abst_detail_view&confID=29&abstractID=10042 | Kobayashi T, et al. Gan To Kagaku Ryoho 2000; 27: 1521-1526 |
|  | Masuyama M, et al. Gan To Kagaku Ryoho 1994; 21: 2253-2255 | Wang XL, et al. Oncol Rep 2000; 7: 241-244 |
|  | Yonemura Y, et al. World J Surg 1993; 17: 256-261; discussion 261-262 | Takiguchi N, et al. Proc Am Soc Clin Oncol 2000; 19: A1178 |
|  | Nishioka B, et al. Gan To Kagaku Ryoho 1982; 9: 1427-1432 | Lygidakis NJ, et al. Hepatogastroenterology 1999; 46: 2035-2038 |
|  |  | Kang YK, et al. Gan To Kagaku Ryoho,1994,21(13):2253-2255 |
|  |  | Masuyama M, et al. Gan To Kagaku Ryoho 1994; 21: 2253-2255 |
|  |  | Yonemura Y, et al. World J Surg 1993; 17: 256-261; discussion 261-262 |
|  |  | Nishioka B, et al. Gan To Kagaku Ryoho 1982; 9: 1427-1432 |

**Supplementary Table S7: Overlap of included studies among meta-analyses regarding surgery with vs. without AC**

| **First author** | **Sun J** | **Sun P** |
| --- | --- | --- |
| Journal (Year) | BMC Cancer (2013) | Br J Surg (2009) |
| Publication type | Full text | Full text |
| No. Included studies | 3 | 12 |
| No. Included RCTs | 0 | 12 |
| Included studies | Saidi RF, et al. World J Surg 2006,30(1):21–27. | Cirera L, et al. J Clin Oncol 1999; 17: 3810–3815. |
|  | Lin SZ, et al. J Cancer Res Clin Oncol 2008, 134(2):187–192. | Nakajima T, et al. Lancet 1999; 354: 273–277. |
|  | Lupascu C, et al. Chirurgia (Bucur) 2010,105(4):473–476. | Neri B, et al. Br J Cancer 2001; 84: 878–880. |
|  |  | Bajetta E, et al. Ann Oncol 2002; 13:299–307. |
|  |  | Nashimoto A, et al. J Clin Oncol 2003; 21: 2282–2287. |
|  |  | Chipponi J, et al.  Am  J Surg 2004; 187: 440–445. |
|  |  | Popiela T, et al. Gastric Cancer 2004; 7: 240–245. |
|  |  | Bouche O, et al. Ann Oncol 2005; 16: 1488–1497. |
|  |  | Nitti D, et al. Ann Oncol 2006; 17: 262–269. |
|  |  | De Vita F, et al. Ann Oncol 2007; 18: 1354–1358. |
|  |  | Nakajima T, et al.  Br J Surg  2007; 94: 1468–1476. |
|  |  | Sakuramoto S, et al. N Engl J Med 2007; 357: 1810–1820. |

**Supplementary Table S8: Overlap of included studies among meta-analyses regarding surgery with vs. without IPC**

| **First author** | **Coccolini F** | **Yan TD** |
| --- | --- | --- |
| Journal (Year) | Eur J Surg Oncol (2014) | Ann Surg Oncol (2007) |
| Publication type | Full text | Full text |
| No. Included studies | 20 | 13 |
| No. Included RCTs | 20 | 13 |
| Included studies | Koga S, et al. Cancer 1988;61:232–7. | Fujimoto S, et al. Cancer 1999; 85:529–34. |
|  | Hagiwara A, et al. Lancet 1992;339:629–31. | Yonemura Y, et al. Hepatogastroenterol 2001; 48:1776–82. |
|  | Hamazoe R, et al. Cancer 1994;73:2048–52. | Hamazoe R, et al. Cancer 1994;73:2048–52. |
|  | Fujimura T, et al. World J Surg 1994;18(1):150–5. | Zhang W, et al. Shanxi Yiyao Zazhi 1998; 27:67–9. |
|  | Sautner T, et al. J Clin Oncol 1994;12(5):970–4. | Miyashiro I, et al. Proc ASCO-GI 84 (abstr 4), 2005. |
|  | Ikeguchi M, et al. Eur J Surg 1995;161:581–6. | Gao Z, et al. Zhonggou Zhongliu Linchuang 2002; 29:294–5. |
|  | Takahashi T, et al. World J Surg 1995;19(4):565–9. | Takahashi T, et al. World J Surg 1995;19(4):565–9. |
|  | Fujimoto S, et al. Cancer 1999;85(3):529–34. | Sautner T, et al. J Clin Oncol 1994; 12:970–4. |
|  | Rosen HR, et al. J Clin Oncol 1998;16(8):2733–8. | Rosen HR, et al. J Clin Oncol 1998;16(8):2733–8. |
|  | Yu W, et al. World J Surg 2001;25(8):985–90. | Yu W, et al. World J Surg 2001;25(8):985–90. |
|  | Shimoyama S, et al. World J Surg 1999;23(3):284–91. | Shimoyama S, et al. World J Surg 1999;23(3):284–91. |
|  | Tan CQ, et al. J Pract Oncol 2000;15(3):165–7. | Zuo Y, et al. Zhonghua Zhongliu Zazhi 2004; 26:247–9. |
|  | Yonemura Y, et al. Hepatogastroenterology 2001;48(42):1776–82. |  |
|  | Wei G, et al. Chinese J Cancer 2005;24(4):478–82. |  |
|  | Zuo Y, et al. Chin J Oncol 2004;26(4):247–9. |  |
|  | Ding WT, et al. Tumor 2007;27(7):585–7. |  |
|  | Kuramoto M, et al. Ann Surg 2009;250:242–6. |  |
|  | Deng HJ, et al. J South Med Univ 2009;29(2):295–7. |  |
|  | Yang XJ, et al. Ann Surg Oncol 2011;18(6):1575–81. |  |
|  | Miyashiro I, et al. Gastric Cancer 2011;14(3):212–8. |  |

**Supplementary Table S9. Overlap of included studies among meta-analyses regarding D2 lymphadenectomy versus D2 with para-aortic nodal dissection (D4)**

| **First author** | **Wang Z** | **Zhang YL** |
| --- | --- | --- |
| Journal (Year) | World J Gastroenterol (2010) | Chinese Journal of General Surgery (2010) |
| Publication type | Full text | Full text |
| No. Included studies | 11 | 4 |
| No. Included RCTs | 7 | 4 |
| Included studies | Sano T, et al. Japan Clinical Oncology Group study 9501. J Clin Oncol 2004;22: 2767-2773 | Jiang BJ, et al. Zhongguo Putong Waike Zazhi 2000; 9: 292-295 |
|  | Sasako M, et al. N Engl J Med 2008; 359: 453-462 | Sasako M, et al. N Engl J Med 2008; 359: 453-462 |
|  | Kulig J, et al. Am J Surg 2007; 193: 10-15 | Kulig J, et al. Am J Surg 2007; 193: 10-15 |
|  | Yonemura Y, et al. Hepatogastroenterology 2006; 53: 389-394 | Yonemura Y, et al. Hepatogastroenterology 2006; 53: 389-394 |
|  | Yonemura Y, et al. Int J Clin Oncol 2008; 13: 132-137 |  |
|  | Jiang BJ, et al. Zhongguo Putong Waike Zazhi 2000; 9: 292-295 |  |
|  | Maeta M, et al. Surgery 1999; 125:325-331 |  |
|  | Hu JK, et al. Surg Today 2009; 39: 207-213 |  |
|  | Kunisaki C, et al. Ann Surg Oncol 2006; 13: 659-667 |  |
|  | Bostanci EB, et al. Eur J Surg Oncol 2004; 30: 20-25 |  |
|  | **Kodera Y**, et al. *Br J Surg* 2005; **92**:1103-1109 |  |

**Supplementary Table S10. Overlap of included studies among meta-analyses regarding chemotherapy vs. basic supportive care (BSC)**

| **First author** | **Badiani B** | **Kim HS** | **Wagner AD** | **Casaretto L** | **Iacovelli R** | **Wagner AD** |
| --- | --- | --- | --- | --- | --- | --- |
| Journal (Year) | World J Clin Oncol (2015) | Ann Oncol (2013) | Cochrane Database Syst Rev (2010) | Braz J Med Biol Res (2006) | PLoS One (2014) | J Clin Oncol (2006) |
| Publication type | Full text | Full text | Full text | Full text | Full text | Full text |
| No. Included studies | 7 | 3 | 3 | 5 | 3 | 3 |
| No. Included RCTs | 7 | 3 | 3 | 5 | 3 | 3 |
| Included studies | Kang JH, et al. J Clin Oncol 2012; 30: 1513-1518 | Kang JH, et al. J Clin Oncol 2012; 30: 1513-1518 | Murad AM, et al. Cancer 1993; 72(1):37–41. | Murad AM, et al. Cancer 1993; 72(1):37–41. | Ford HE, et al. Lancet Oncol 2012;15: 78–86 | Murad AM, et al. Cancer 1993; 72(1):37–41. |
|  | Hironaka S, et al. J Clin Oncol 2013; 31: 4438-4444 | Cook N, et al. J Clin Oncol 2013; 31 (suppl): abstract 4023 | Pyrhonen S, et al. British Journal of Cancer 1995; 71(3):587–91. | Pyrhonen S, et al. British Journal of Cancer 1995; 71(3):587–91. | Thuss-Patience PC, et al. Eur J Cancer 2011;47: 2306–14 | Pyrhonen S, et al. British Journal of Cancer 1995; 71(3):587–91. |
|  | Thuss-Patience PC, et al. Eur J Cancer 2011; 47: 2306-2314 | Thuss-Patience PC, et al. Eur J Cancer 2011; 47: 2306-2314 | Scheithauer W, et al. Annals of Hematology. 1996; 73 (Suppl 2):A181. | Dent DM, et al. Cancer 1979; 44: 385-391. | Kang JH, et al. J Clin Oncol 2012;30: 1513–8 | Scheithauer W, et al. Annals of Hematology. 1996; 73 (Suppl 2):A181. |
|  | Ford HE, et al. Lancet Oncol 2014; 15: 78-86 |  |  | Glimelius B, et al. Annals of Oncology 1997; 8:163-168. |  |  |
|  | Ohtsu A, et al. J Clin Oncol 2013; 31: 3935-3943 |  |  | Kingston RD, et al. Clinical  Oncology 1978; 4: 55-69 |  |  |
|  | Fuchs CS, et al. Lancet 2014; 383:31-39 |  |  |  |  |  |
|  | Wilke H, et al. Lancet Oncol 2014; 15: 1224-1235 |  |  |  |  |  |

**Supplementary Table S11. Overlap of included studies among meta-analyses regarding S-1-based therapy vs. 5-FU-based therapy**

| **First author** | **Ter Veer E** | **Wu FL** | **Yang J** | **Li DH** | **Liu H** | **Huang J** |
| --- | --- | --- | --- | --- | --- | --- |
| Journal (Year) | Gastric Cancer (2016) | Medicine (Baltimore) (2015) | World J Gastroenterol (2014) | Tumour Biol (2014) | Medicine (Baltimore) (2014) | Med Oncol.  (2011) |
| Publication type | Full text | Full text | Full text | Full text | Full text | Full text |
| No. Included studies | 8 | 6 | 4 | 6 | 3 | 4 |
| No. Included RCTs | 8 | 6 | 4 | 6 | 3 | 4 |
| Included studies | Jin M, et al. J Clin Oncol. 2008;26(15 Suppl):4533 | Jin M, et al. J Clin Oncol. 2008;26:4533 | Ajani JA, et al.  Eur J Cancer 2013; 49:  3616-3624 | Jin M, et al. J Clin Oncol. 2008;26:4533 | Han XH, et al Chinese J Dis Control Prevent. 2012;16:974–978 | Jin M, et al. J Clin Oncol. 2008;26:4533 |
|  | Boku N, et al. Lancet Oncol. 2009;10(11):1063–69. | Boku N, et al. Lancet Oncol. 2009;10:1063–1069 | Boku N, et al. Lancet Oncol. 2009;10(11):1063–69. | Boku N, et al. Lancet Oncol. 2009;10:1063–1069 | Nishikawa K, et al. Gastric Cancer. 2012;15:363–369 | Boku N, et al. Lancet Oncol. 2009;10(11):1063–69. |
|  | Ajani JA, et al. J Clin Oncol. 2010;28(9):1547–53. | Ajani JA, et al. J Clin Oncol. 2010;28:1547–1553 | Huang DZ, et al. Eu J Cancer (Oxford, England: 1990). 2013;49:2995–3002 | Ajani JA, et al. J Clin Oncol. 2010;28:1547–1553 | Huang DZ, et al. Eu J Cancer (Oxford, England: 1990). 2013;49:2995–3002 | Ajani JA, et al. Gastrointestinal Cancers Symposium. 2009. |
|  | Nishikawa K, et al. Gastric Cancer. 2012;15(4):363–9. | Nishikawa K, et al. Gastric Cancer. 2012;15:363–369 | Nishikawa K, et al. Gastric Cancer. 2012;15:363–369 | Nishikawa K, et al. Gastric Cancer. 2012;15:363–369 |  | Fuse N, et al. J Clin Oncol. 2009;27:15s |
|  | Xu RH, et al. J Clin Oncol. 2013;31:4025 | Xu RH, et al. J Clin Oncol. 2013;15(Suppl):11 |  | Xu RH, et al. J Clin Oncol. 2013;15(Suppl):11 |  |  |
|  | Huang DZ, et al. Eu J Cancer (Oxford, England: 1990). 2013;49:2995–3002 | Huang DZ, et al. Eu J Cancer (Oxford, England: 1990). 2013;49:2995–3002 |  | Huang DZ, et al. Eu J Cancer (Oxford, England: 1990). 2013;49:2995–3002 |  |  |
|  | Sawaki A, et al. EJC Suppl.  2009;7(2):364 |  |  |  |  |  |
|  | Ajani JA, et al. J Clin Oncol. 2015;33(15 Suppl):4015 |  |  |  |  |  |

**Supplementary Table S12. Overlap of included studies among meta-analyses regarding S-1-based therapy vs. capecitabine based therapy**

| **First author** | **Ter Veer E** | **Wu FL** | **Yang J** | **He MM** |
| --- | --- | --- | --- | --- |
| Journal (Year) | Gastric Cancer (2016) | Medicine (Baltimore) (2015) | World J Gastroenterol (2014) | PLoS One (2013) |
| Publication type | Full text | Full text | Full text | Full text |
| No. Included studies | 3 | 8 | 2 | 10 |
| No. Included RCTs | 3 | 5 | 2 | 5 |
| Included studies | Lee JL, et al. Br J Cancer. 2008;99(4):584–90 | Lee JL, et al. Br J Cancer. 2008;99(4):584–90 | Lee JL, et al. Br J Cancer. 2008;99(4):584–90 | Lee JL, et al. Br J Cancer. 2008;99(4):584–90 |
|  | Kim GM, et al. Eur J Cancer. 2012;48(4):518–26 | Kim GM, et al. Eur J Cancer. 2012;48(4):518–26 | Kim GM, et al. Eur J Cancer. 2012;48(4):518–26 | Kim GM, et al. Eur J Cancer. 2012;48(4):518–26 |
|  | Kobayashi M, et al. J Clin Oncol. 2015;33(3 Suppl):105 | Shitara K, et al. Int J Clin Oncol. 2013;18:539–546. |  | Shitara K, et al. Int J Clin Oncol. 2013; 18: 539-546. |
|  |  | Seol YM, et al. Jpn J Clin Oncology. 2009;39:43–48 |  | Seol YM, et al. Jpn J Clin Oncology. 2009;39:43–48 |
|  |  | Ba Z, et al. Chinese General Pract.2012;15:672–676. |  | Ba Z, et al. Chinese General Pract.2012;15:672–676. |
|  |  | Yan Z, et al. Progress Mod Biomed. 2012;12:5324–5326 |  | Yan SN. Chin J Gerontol. 2012;32:2382-2383. |
|  |  | Lu HF, et al. China Med Herald.2012;9:61–63 |  | Lu HF, et al. China Med Herald.2012;9:61–63 |
|  |  | Xiong HL, et al. Med Oncol (Northwood, London, England). 2013;21:581–584 |  | Xiong HL, et al. Med Oncol (Northwood, London, England). 2013;21:581–584 |
|  |  |  |  | Gao W, et al. Pract Geriatr. 2012;26: 501-504 |
|  |  |  |  | Lim D, et al. BMC Cancer. 2010;10: 583-589. |

**Supplementary Table S13. Overlap of included studies among meta-analyses regarding S-1-based combination therapy vs. S-1 monotherapy**

| **First author** | **Ter Veer E** | **Liu GF** | **Wu JR** |
| --- | --- | --- | --- |
| Journal (Year) | Gastric Cancer (2016) | World J Gastroenterol (2014) | Tumour Biol (2014) |
| Publication type | Full text | Full text | Full text |
| No. Included studies | 8 | 4 | 6 |
| No. Included RCTs | 8 | 4 | 5 |
| Included studies | Koizumi W, et al. Lancet Oncol. 2008;9(3):215–21 | Koizumi W, et al. Lancet Oncol. 2008;9(3):215–21 | Koizumi W, et al. Lancet Oncol. 2008;9(3):215–21 |
|  | Komatsu Y, et al. Anticancer Drugs. 2011;22(6):576–83 | Komatsu Y, et al. Anticancer Drugs. 2011;22(6):576–83 | Komatsu Y, et al. Anticancer Drugs. 2011;22(6):576–83 |
|  | Narahara H, et al. Gastric  Cancer. 2011;14(1):72–80 | Narahara H, et al. Gastric  Cancer. 2011;14(1):72–80 | Narahara H, et al. Gastric  Cancer. 2011;14(1):72–80 |
|  | Wang X, et al. Clin Transl Oncol. 2013;15(10):836–42 | Wang X, et al. Clin Transl Oncol. 2013;15(10):836–42 | Wang X, et al. Clin Transl Oncol. 2013;15(10):836–42 |
|  | Lu Y, et al. J Chemother. 2014;26(3):159–64 |  | Lai LN, et al. China Med Her. 2012;9:28–31 |
|  | Jin M, et al. J Clin Oncol. 2008;26(15 Suppl):4533 |  | Tsushima T, et al. Int J Clin Oncol. 2013;18:10–6 |
|  | Koizumi W, et al. J Cancer Res Clin Oncol. 2014;140(2):319–28 |  |  |
|  | Yamaguchi K, et al. Ann  Oncol. 2014;25(Suppl 4):iv210–253. |  |  |

**Supplementary Table S14. Overlap of included studies among meta-analyses regarding oxaliplatin vs. cisplatin**

| **First author** | **Montagnani F** | **Wagner AD** | **Gong JF** |
| --- | --- | --- | --- |
| Journal (Year) | Gastric Cancer (2011) | Cochrane Database Syst Rev (2010) | Zhonghua Yi Xue Za Zhi (2009) |
| Publication type | Full text | Full text | Full text |
| No. Included studies | 3 | 2 | 16 |
| No. Included RCTs | 3 | 2 | NA |
| Included studies | Al-Batran SE, et al. J Clin Oncol. 2008;26(9):1435–42 | Al-Batran SE, et al. J Clin Oncol. 2008;26(9):1435–42 | Zeng HM. Chinese Medical of Factory and Mine, 2003,16;451-452 |
|  | Popov I, et al. J BUON. 2008;13:505–11 | Popov I, et al. J BUON. 2008;13:505–11 | Popov I, et al. J BUON, 2008;13:505–11 |
|  | Cunningham D, et al. N Engl J Med. 2008;358:36–46. |  | Pan RQ, et al. Sichuan Journal of Cancer, 2005,18;227-234 |
|  |  |  | Feng LY, et al. Chinese Journal of Clinical Oncology and Rehabilitation, 2006,13;503-505 |
|  |  |  | Tang DX, et al. Journal of Basic and Clinical Oncology, 2006,13:126-127 |
|  |  |  | Zhou GQ, et al. Chinese Journal of Misdiagnostics, 2006,6:98-99 |
|  |  |  | Wu P, et al. Chinese Journal of Cancer Prevention and Treatment, 2006,13:58-60 |
|  |  |  | Ye SC, et al. Clinical Focus, 2005,20:1150-1152 |
|  |  |  | Tian WW, et al. Journal of Medical Forum, 2007,28:13-14 |
|  |  |  | Wang HM, et al. China Journal of Modern Medicine, 2006,16:2626-2628 |
|  |  |  | Xu XJ, et al. Journal of Clinical Medicine in Practice, 2004,8:52-54 |
|  |  |  | Wang QC, et al. Chinese Journal of Misdiagnostics, 2006,6:2510-2511 |
|  |  |  | Fang YS, et al. Chinese Clinical Oncology, 2004,9:537-539 |
|  |  |  | Salah-Eddin A, et al. J Clin Oncol, 2008,26:1435-1442 |
|  |  |  | David C, et al. N Eng J Med, 2008,358:36-39 |
|  |  |  | Xie Z, et al. Chinese Journal of Clinical Oncology, 2006,33:1041-1046 |

**Supplementary Table S15. Overlap of included studies among meta-analyses regarding capecitabine vs. 5-FU**

| **First author** | **Xu HB** | **Wagner AD** | **Ma Y** |
| --- | --- | --- | --- |
| Journal (Year) | Eur J Clin Pharmacol (2015) | Cochrane Database Syst Rev (2010) | J Clin Pharm Ther (2012) |
| Publication type | Full text | Full text | Full text |
| No. Included studies | 26 | 1 | 18 |
| No. Included RCTs | 26 | 1 | 18 |
| Included studies | Sun Q, et al. Cancer Res Prev Treat 2005,32:729–730 | Kang YK, et al. Annals of Oncology, 2009;20:666–673 | Sun Q, et al. Cancer Res Prev Treat 2005,32:729–730 |
|  | Cai DY, et al. J Clin Exp Med 2007,16:45–46 |  | Cai DY, et al. J Clin Exp Med 2007,16:45–46 |
|  | Chen SJ, et al. China Oncol 2007,17:483–486 |  | Chen SJ, et al. China Oncol 2007,17:483–486 |
|  | Qu ZF, et al. Fujian Med J 2007,29:41–43 |  | Qu ZF, et al. Fujian Med J 2007,29:41–43 |
|  | Xue FQ, et al. Fujian Med J 2008,30:114–116 |  | Xue FQ, et al. Fujian Med J 2008,30:114–116 |
|  | Shi M, et al. J South Med Univ 2008,28:1490–1491 |  | Shi M, et al. J South Med Univ 2008,28:1490–1491 |
|  | Gao X, et al. Mod Med J China 2008,10:35–37 |  | Wang L, et al. Modern Oncology, 2005;13:361–362 |
|  | Wang HM, et al. Eval Anal Drug-Use Hosp China 2009,9:856–857 |  | Jia F, et al. Exp Clin Oncol, 2008;20:276–278 |
|  | Hu JB, et al. China Pract Med 2009,4:25–26 |  | Hu JB, et al. China Pract Med 2007,29:25–26 |
|  | Zhao WY, et al. Chin J Clin  Oncol 2009,36:1044–1046 |  | Zhao WY, et al. Chin J Clin Oncol 2009,36:1044–1046 |
|  | Lei JH, et al. Med J Natl  Defending Forces N China 2009,21:10–12 |  | Lei JH, et al. Med J Natl Defending Forces N China 2009,21:10–12 |
|  | Cui WF, et al. Chin J Curr Adv Gen Surg 2009,12:869–871 |  | Wang JX. Henan Med Res, 2009;18:207–209 |
|  | Wang HZ, et al. Mod Oncol 2010,18:947–950 |  | Wang HZ, et al. Mod Oncol 2010,18:947–950 |
|  | Du C, et al. Inn Mong Med J 2010,42:260–263 |  | Du C, et al. Inn Mong Med J 2010,42:260–263 |
|  | Liu C, et al. Chin J Curr  Adv Gen Surg 2010,13:960–963 |  | Chen SR. Journal of Modern Combination of Traditional Chinese Western  Medicine, 2009;18:3708–3709 |
|  | Liu FL, et al. J Clin Med Pract 2010,14:50–51 |  | Lee RC, et al. Journal of Clinical Internal Medicine, 2008;30:  821–823 |
|  | Wu F, et al. Chin J Clin Gastroenterol 2011,23:  330–331 |  | Kang YK, et al. Annals of Oncology, 2009;20:666–673 |
|  | Lu HL, et al. J Clin Med Pract 2011,15:112–113 |  | Cunningham D, et al. New England Journal of Medicine, 2008;358:36–46 |
|  | Wang L, et al. Anhui Med Pharm J 2011,15:329–330 |  |  |
|  | Yang J. Pract J Card Cereb Pneumal Vasc Dis 2011,19:369–370 |  |  |
|  | Jiang ZH. Guide China Med 2012,10:  118–119 |  |  |
|  | Zhou JH, et al. Chin Foreign Med Res 2012,10:11–12 |  |  |
|  | Wang AY, et al. China Med Her 2012,9:58–59 |  |  |
|  | Hu ZH, et al. Chongqing Med 2013,42:156–159 |  |  |
|  | Fan ZM, et al. Pract J Cancer 2013,28:396–398 |  |  |
|  | Zhang D. Chin J Med Guid 2014,16:129–130 |  |  |

**Supplementary Table S16. Overlap of included studies among meta-analyses regarding irinotecan (CPT-11)-based therapy vs. non CPT-11 therapy**

| **First author** | **Qi WX** | **Wang DL** | **Wagner AD** | **Wagner AD** |
| --- | --- | --- | --- | --- |
| Journal (Year) | Int J Cancer (2013) | World J Gastroenterol (2010) | Cochrane Database Syst Rev (2010) | J Clin Oncol (2006) |
| Publication type | Full text | Full text | Full text | Full text |
| No. Included studies | 10 | 4 | 4 | 3 |
| No. Included RCTs | 10 | 4 | 4 | 3 |
| Included studies | Hawkins R,et al. Proc Am Soc Clin Oncol 2003;22: Abstract 1032. | Dank M, et al. Ann Oncol 2008; 19: 1450-1457 | Dank M, et al. Ann Oncol 2008; 19: 1450-1457 | Dank M, et al. J Clin Oncol 23: 308s, 2005 (suppl 16, abstr 4003) |
|  | Bouche O,et al. J Clin Oncol 2004;22:4319–28. | Bouche O,et al. J Clin Oncol 2004;22:4319–28. | Bouche O,et al. J Clin Oncol 2004;22:4319–28. | Bouche O,et al. J Clin Oncol 2004;22:4319–28. |
|  | Moehler M, et al. Br J Cancer 2005;92:2122–8 | Moehler M, et al. Br J Cancer 2005;92:2122–8 | Moehler M, et al. Br J Cancer 2005;92:2122–8 | Moehler M, et al. Br J Cancer 2005;92:2122–8 |
|  | Nakashima K, et al. Jpn J Clin Oncol 2008;38:810–15 | Nakashima K, et al. Jpn J Clin Oncol 2008;38:810–15 | Moehler MH, et al. Proceedings/Annual Meeting of the American Society of Clinical Oncology. 2004; Vol. 23:328. |  |
|  | Boku N, et al. Lancet 2009;10:1063–9. |  |  |  |
|  | Boukovinas I, et al. J Clin Oncol 2009;27: Abstract 4536. |  |  |  |
|  | Kishimoto T, et al. J Clin Oncol 2010;28: Abstract 4015 |  |  |  |
|  | Komatsu Y, et al. Anticancer Drugs 2011;22:576–83. |  |  |  |
|  | Narahara H, et al. Gastric Cancer 2011;14:72–80. |  |  |  |
|  | Glenjen N, et al. J Clin Oncol 2012;30: Abstract 71. |  |  |  |

**Supplementary Table S17. Overlap of included studies among meta-analyses regarding targeted therapy**

| **First author** | **Ciliberto D** | **Iacovelli R** | **Qi WX** |
| --- | --- | --- | --- |
| Journal (Year) | Cancer Biol Ther (2015) | PLoS One (2014) | Tumour Biol (2014) |
| Publication type | Full text | Full text | Full text |
| No. Included studies | 22 | 2 | 7 |
| No. Included RCTs | 22 | 2 | 7 |
| Included studies | Fuchs CS, et al. Lancet 2014; 383:31-9 | Fuchs CS, et al. Lancet 2014; 383:31-9 | Fuchs CS, et al. Lancet 2014; 383:31-9 |
|  | Ohtsu A, et al. J Clin Oncol 2013; 31:3935-43 | Ohtsu A, et al. J Clin Oncol 2013; 31:3935-43 | Wilke H, et al. ASCO Meet Abstr 2014;32:LBA7 |
|  | Koizumi W, et al. Br J Cancer 2013;109:2079-86 |  | Koizumi W, et al. Br J Cancer 2013;109:2079-86 |
|  | Richards D, et al. Eur J Cancer 2013;49:2823-31 |  | Yi JH, et al. Br J Cancer 2012;106:1469-74 |
|  | Waddell T, et al. Lancet Oncol 2013; 14:481-9 |  | Li J, et al. J Clin Oncol 2013; 31:3219-25 |
|  | Bang YJ, et al. Lancet 2010; 376:687-97; PMID:20728210 |  | Ohtsu A, et al. J Clin Oncol 2011; 29:3968-76 |
|  | Ohtsu A, et al. J Clin Oncol 2011; 29:3968-76 |  | Shen L, et al. Gastric cancer : official  journal of the International Gastric Cancer Association and  the Japanese Gastric Cancer Association 2014 |
|  | Hecht, et al. ASCO Meet Abstr 2013; 31:LBA4001 |  |  |
|  | Lordick F, et al. Lancet Oncol 2013;14:490-9 |  |  |
|  | Cohen DJ, et al. ASCO Meet Abstr 2013; 31:4011 |  |  |
|  | Iveson T, et al. Lancet Oncol 2014; 15:1007-18 |  |  |
|  | Yoon HH, et al. ASCO Meet Abstr 2014; 32:4004 |  |  |
|  | Wilke H, et al. ASCO Meet Abstr 2014;32:LBA7 |  |  |
|  | Bang Y-J. ASCO Meet Abstr 2013; 31:11 |  |  |
|  | Bang Y-J, et al. ASCO Meet Abstr 2013;31:4013 |  |  |
|  | Moehler MH, et al. ASCO Meet Abstr 2010; 28: e14503 |  |  |
|  | Kim YH, et al. ASCO Meet Abstr 2011; 29:87 |  |  |
|  | Yi JH, et al. Br J Cancer 2012;106:1469-74 |  |  |
|  | Li J, et al. J Clin Oncol 2013; 31:3219-25 |  |  |
|  | Rao S, et al. Ann Oncol 2010; 21:2213-9 |  |  |
|  | Eatock MM, et al. Ann Oncol 2013; 24:710-8 |  |  |
|  | Qin S. ASCO Meet Abstr 2014; 32:4003 |  |  |

**Supplementary Table S18. Overlap of included studies among meta-analyses regarding combination (doublet/triplet) therapy vs. single/doublet therapy**

| **First author** | **Zhang Y** | **Liu N** | **Wagner AD** | **Wagner AD** |
| --- | --- | --- | --- | --- |
| Journal (Year) | Medicine (Baltimore) (2016) | Chinese Journal of Hospital Pharmacy (2012) | Cochrane Database Syst Rev (2010) | J Clin Oncol (2006) |
| Publication type | Full text | Full text | Full text | Full text |
| No. Included studies | 10 | 12 | 13 | 11 |
| No. Included RCTs | 10 | 12 | 13 | 11 |
| Included studies | Maruta F, et al. Ann Oncol. 2015;26:1916–1922 | Van Cutsem E, et al. J Clin Oncol, 2006,24(31):4991-4997 | Cullinan SA, et al. JAMA 1985;253:2061–7. | Cullinan SA, et al. JAMA 1985;253:2061–7. |
|  | Sym SJ, et al. Cancer Chemother Pharmacol. 2013;71:481–488 | Ajani JA, et al. J Clin Oncol, 2005,23(24):5660-5667 | Levi JA, et al. Journal of Clinical Oncology 1986;4(9):1348–55 | Levi JA, et al. Journal of Clinical Oncology 1986;4(9):1348–55 |
|  | Ueda A, et al. Anticancer Res. 2013;33:5107–5111 | Roth AD, et al. J Clinical Oncol, 2007,25(22):3217-3223 | De Lisi V, et al. Cancer Treatment Reports 1986;70:481–5. | De Lisi V, et al. Cancer Treatment Reports 1986;70:481–5. |
|  | Satoh T, et al. J Clin Oncol. 2014;32:2039–2049 | Xiao-Dong Li, et al. World J Gastroenterol, 2011,17(8):1082-1087 | Cullinan SA, et al. Journal of Clinical Oncology 1994;12(2):412–6. | Cullinan SA, et al. Journal of Clinical Oncology 1994;12(2):412–6. |
|  | Wilke H, et al. Lancet Oncol. 2014;15:1224–1235. | Kyoto Research Group for Chemotherapy of Gastric Cancer. Anticancer Res, 1992,12:1983-1988 | Barone C, et al. Cancer 1998;82(8):1460–7 | Barone C, et al. Cancer 1998;82(8):1460–7 |
|  | Kim HS, et al. Ann Oncol. 2013;24:2850–2854. | Kim TW, et al. Eur J Cancer, 2001,37 (Suppl 6):314 | Yamamura Y, et al. Japanese  Journal of Cancer & Chemotherapy 1998;25(10):1543–8. | Yamamura Y, et al. Japanese  Journal of Cancer & Chemotherapy 1998;25(10):1543–8. |
|  | Lorenzen S, et al. Eur J Cancer. 2015;51:569–576 | Koizumi W, et al. Anticancer Res, 2004,24:2465-2470 | Popov I, et al. Annals of Oncology. 2002; Vol. 13 (Suppl. 5):188. | Popov I, et al. Annals of Oncology. 2002; Vol. 13 (Suppl. 5):188. |
|  | Nishikawa K, et al. Eur J Cancer. 2015;51:808–816. | Thuss-Patience PC, et al. J Clinical Oncol, 2005,23(3):494-501 | Ohtsu A, et al. Journal of Clinical  Oncology 2003;21(1):54–9 | Ohtsu A, et al. Journal of Clinical  Oncology 2003;21(1):54–9 |
|  | Satoh T, et al. Gastric Cancer. 2015;18:824–832. | Yun J, et al. Eur J Cancer, 2010,46:885-897 | Bouche O, et al. Journal of Clinical Oncology 2004;22:4319–29. | Bouche O, et al. Journal of Clinical Oncology 2004;22:4319–29. |
|  | Tanabe K, et al. Ann Oncol. 2015;26:1916–1922 | Roth A, et al. Tumori, 1999,85(4):234-238 | Lutz MP, et al. Journal of Clinical Oncology 2007;25(18):2580–5. | Colucci G, et al. Am J Clin Oncol 18:519-524, 1995 |
|  |  | Vanhoefer U, et al. J Clin Oncol, 2000,18(14):2648-2657 | Koizumi W, et al. Lancet Oncology 2008;9(3):215–21. | Loehrer PJ Sr, et al. Investigational New Drugs 1994;12(1):57–63 |
|  |  | Park SH, et al. Annals Oncol, 2008,19:729-733 | Colucci G, et al. European Journal of  Cancer. 1991; Vol. 27 Suppl. 2:S81. |  |
|  |  |  | Loehrer PJ Sr, et al. Investigational New Drugs 1994;12(1):57–63 |  |

**Supplementary Table S19. Overlap of included studies among meta-analyses regarding FU/anthracycline-containing combinations with vs. without cisplatin**

| **First author** | **Wagner AD** | **Wagner AD** |
| --- | --- | --- |
| Journal (Year) | J Clin Oncol (2006) | Cochrane Database Syst Rev (2010) |
| Publication type | Full text | Full text |
| No. Included studies | 7 | 7 |
| No. Included RCTs | 7 | 7 |
| Included studies | Gastrointestinal Tumor Study Group. J Natl Cancer Inst 1988;80:1011-1015 | Gastrointestinal Tumor Study Group. Journal of the National Cancer Institute 1988;80:1011–5. |
|  | Coccioni G, et al. J Clin Oncol 1994;12:2687-2693 | Cocconi G, et al. Journal of Clinical Oncology 1994;12(12):2687–93 |
|  | Kikuchi K, et al. Gan To Kagaku Ryoho 1990;17:655-662 | Kikuchi K, et al. Gan to Kagaku Ryoho 1990;17(4 Pt 1):655–62 |
|  | Cullinan SA, et al. Journal of Clinical Oncology 1994;12(2):412–6 | Cullinan SA, et al. Journal of Clinical Oncology 1994;12(2):412–6. |
|  | Webb A, et al. J Clin Oncol 1997;15:261-267, | Webb A, et al. Journal of Clinical Oncology 1997;15(1):261–7 |
|  | Roth A, et al. Tumori 1999;85:234-238, | Roth A, et al. Tumori 1999;85(4):234–8 |
|  | Coccioni G, et al. Ann Oncol 2003;14:1258-1263 | Cocconi G, et al. Annals of Oncology 2003;14(8):1258–63 |

**Supplementary Table S20. Overlap of included studies among meta-analyses regarding FU/cisplatin-containing regimens with vs. without anthracyclines**

| **First author** | **Wagner AD** | **Wagner AD** |
| --- | --- | --- |
| Journal (Year) | Cochrane Database Syst Rev (2010) | J Clin Oncol (2006) |
| Publication type | Full text | Full text |
| No. Included studies | 3 | 3 |
| No. Included RCTs | 3 | 3 |
| Included studies | Kyoto Research Group for Chemotherapy of Gastric Cancer. Anticancer Research 1992;12:1983–8 | Kyoto Research Group for Chemotherapy of Gastric Cancer. Anticancer Research 1992;12:1983–8 |
|  | Ross P, et al. Journal of Clinical Oncology 2002;20(8):1996–2004. | Ross P, et al. Journal of Clinical Oncology 2002;20(8):1996–2004. |
|  | Kim TW, et al. European Journal of Cancer 2001;37(Suppl 6):314 | Kim TW, et al. European Journal of Cancer 2001;37(Suppl 6):314 |

**Supplementary Table S21. Overlap of included studies among meta-analyses regarding Shenqifuzheng injection plus chemotherapy vs. chemotherapy alone**

| **First author** | **Li J** | **Yao K** |
| --- | --- | --- |
| Journal (Year) | Chin J Integr Med (2015) | J Cancer Res Ther (2014) |
| Publication type | Full text | Full text |
| No. Included studies | 13 | 15 |
| No. Included RCTs | 13 | NA. |
| Included studies | Liu H, et al. J Chin Pharmacol 2011;27:177-83 | Liu H, et al. J Chin Pharmacol 2011;27:177-83 |
|  | Jia JW, et al. Pract J Cancer 2009;3:273-5 | Jia JW, et al. Pract J Cancer 2009;3:273-5 |
|  | Zhou KX, et al. Chin J Integr Trad Western Med 1999;1:1-6 | Zhou KX, et al. Chin J Integr Trad Western Med 1999;1:1-6 |
|  | Xiong W. Guangming J Chin Med 2010;25:635-6 | Xiong W. Guangming J Chin Med 2010;25:635-6 |
|  | Wan LX, et al. J Med Forum 2006;27:96-7 | Wan LX, et al. J Med Forum 2006;27:96-7 |
|  | Li ZY, et al. J Liaoning Univ Trad Chin Med 2006;8:89-90 | Li ZY, et al. J Liaoning Univ Trad Chin Med 2006;8:89-90 |
|  | Zhao JM, et al. Chin J Integr Med 2007;27:736-8 | Zhao JM, et al. Chin J Integr Med 2007;27:736-8 |
|  | Wang JF. Chin Med Sci Technol (Chin) 2010;17:537-538. | Chen M, et al. Chin Commun Doct 2010;12:113-4 |
|  | Fang XY. Chin J Pract Med 2010;37:23-4 | Fang XY. Chin J Pract Med 2010;37:23-4 |
|  | Wang YS, et al. Med J West Chin (Chin) 2011;23:1958-1959 | Zhang WH, et al. J Baotou Med Coll 2012;28:86-7 |
|  | Wang M, et al. Chin Modern Med 2011;18:89-90 | Wang M, et al. Chin Modern Med 2011;18:89-90 |
|  | Luo PF. Modern Med Health 2011;27:1170-1 | Luo PF. Modern Med Health 2011;27:1170-1 |
|  | Yue CL. Chin New Med (Chin) 2003;4:1063-1064 | Pa TM, et al. World Latest Med Inform 2012:46-7 |
|  |  | Ren YZ, et al. J Basic Clin Oncol 2012;25:394-6 |
|  |  | Zhu LF, et al. Shanghai Med J 2007;30:542-3. |
